# Integrated Early Warning Surveillance: Achilles′ Heel of One Health?

**DOI:** 10.3390/microorganisms8010084

**Published:** 2020-01-08

**Authors:** Laura Amato, Maria Grazia Dente, Paolo Calistri, Silvia Declich, on behalf of the MediLabSecure Working Group

**Affiliations:** 1 National Centre for Global Health, Istituto Superiore di Sanità, 00161 Rome, Italy; laura.amato@iss.it (L.A.); silvia.declich@iss.it (S.D.); 2 Public Health and Infectious Diseases Department, Sapienza University of Rome, 00185 Rome, Italy; 3 National Reference Center for Veterinary Epidemiology, Istituto Zooprofilattico Sperimentale dell’Abruzzo e del Molise ′G.Caporale′, 64100 Teramo, Italy; p.calistri@izs.it

**Keywords:** arbovirus, early warning, integrated surveillance, One Health

## Abstract

Emerging and re-emerging infectious diseases and zoonoses indicate the importance of the One Health (OH) approach for early warning. At present, even when surveillance data are available, they are infrequently timeously shared between the health sectors. In the context of the MediLabSecure (MLS) Project, we investigated the collection of a set of surveillance indicators able to provide data for the implementation of integrated early warning systems in the 22 MLS countries of the Mediterranean, Black Sea and Sahel regions. We used an online questionnaire (covering vector, human, and animal sectors), focusing on seven relevant arboviruses, that was submitted to 110 officially appointed experts. Results showed that West Nile virus was perceived as the most relevant zoonotic pathogen, while Dengue virus was the most relevant non-zoonotic pathogen in the study area. Data collection of early warning indicators is in place at a different level for all the investigated pathogens and in almost all the MLS Countries. Further assessments on the reliability of the collection in place and on the feasibility of piloting an integrated early warning system for arbovirus could verify if integrated early warning really represents the Achilles’ heel of OH.

## 1. Introduction

The One Health (OH) concept is gathering greater attention over the last decades due to the (re)emergence of human pathogens from animal reservoirs and the outcomes of studies on the impact of environmental and climate changes on the transmission of several infectious diseases [1,2,3,4]. OH is defined as a collaborative, multi-sectoral, and trans-disciplinary approach to optimizing health of people, animals, and the shared environment [5]. At the global level, the World Organization for Animal Health (OIE), the World Health Organization (WHO), and the Food and Agriculture Organization of the United Nations (FAO) officially endorsed OH collaborations through a tripartite agreement and a guide addressing zoonotic diseases [6,7].

The OH approach is promising in terms of surveillance and control of vector-borne diseases (VBDs) because it makes use of trans-disciplinary cooperation at the human–animal–ecosystem interfaces [8,9]. Among VBDs, arboviruses have complex life cycles involving both human and animal hosts and, being transmitted by arthropod vectors, are closely linked to environmental and climatic conditions [10]. Therefore, surveillance of VBD is one of the best examples of diseases benefiting from the establishment of integrated systems in accordance with the OH concept [11,12,13,14].

With limitations, integrated surveillance (“One Health surveillance describes the systematic collection, validation, analysis, interpretation of data and dissemination of information collected on humans, animals and the environment to inform decisions for more effective, evidence- and system-based health interventions” [9]) systems for arbovirus infections have been implemented in a number of countries [8,15,16]. It appears, however, that in the majority of the countries, even when the different sectors involved (i.e., human, animal, entomological, and environmental) collect surveillance data, rarely is this information shared in a timely manner between sectors to prevent outbreaks. An early-warning capacity is therefore weak or lacking, and needs to be reinforced [15,17].

In fact, the identification of early warning indicators in association with rapid implementation of prevention and control measures could reduce the severity of arbovirus epidemics [18,19]. Ad hoc indicators can also highlight the vulnerability of countries or specific zones to the introduction and spread of arbovirus infections, thus providing precious information to prevent the occurrence of outbreaks and epidemics [20,21].

To tackle this, a number of studies have already been performed to predict the risk of VBDs transmission using climate data [22,23,24,25], sectorial information from animals [26,27], from humans [28,29,30], and from vectors [31,32,33].

This subject is crucial in the networking project named MediLabSecure (MLS), which involves 22 non-EU countries of the Mediterranean, Black Sea, and Sahel regions [34]. The network is focused on strengthening integrated surveillance of arbovirus infections, given that the use of early warning data is still considered “pioneeristic” in the MLS region [8].

To this aim, we identified a set of surveillance indicators that could assist in increasing regional early-warning capacity and verified, through a survey, their collection at the national level.

## 2. Materials and Methods

The study focused on emerging and re-emerging arboviruses (Crimean-Congo haemorrhagic fever virus–CCHFV, Chikungunya virus—CHIKV, Dengue virus—DENV, Rift Valley fever virus—RVFV, West Nile virus—WNV, Yellow fever virus—YFV, and Zika virus—ZIKV) representing possible priorities for the various geographical areas included in MLS network. Moreover, general interest to these pathogens was confirmed in late 2018 during a OH scientific conference held in Rome [35] and a OH workshop organized in Teramo [36] with representatives (i.e., public health and veterinarian officers working at country level who were officially appointed to represent their institutions in the MLS network) of the countries involved in the network. They highlighted, among gaps and needs, that some of the surveillance systems currently in place were not adequate, and scarcity of integration between the involved systems persists.

From January 2019, using scientific references [10,20,21,26,37] and grey literature [38,39,40], we identified possible indicators for highlighting vulnerabilities of MLS countries to the selected arboviral diseases. A set (from four to six) of potential indicators were identified for each pathogen and sector involved in arbovirus surveillance (entomology, human health, and, in the case of zoonotic pathogens, animal health). MLS experts in entomology, human and animal virology, public health, and animal health reviewed the selected indicators and we finalized the list.

In order to gather local information about the utilization of potential indicators in the countries of the MLS network, we developed an online questionnaire using Google Form©. A draft form of the questionnaire was piloted by a small group of external experts who assessed appropriateness of wording, comprehensibility, clarity of the instructions, and time needed to complete the questionnaire.

We implemented the questionnaire in three versions (for vector, human, and animal sectors), harbouring common features and sector-specific questions for each pathogen (see Supplementary Materials). At the beginning of each section, we asked about the relevance of the pathogen for the specific country; a definition of “relevance” was given as follows: “an endemic or epidemic pathogen in the country, or an emerging pathogen not yet identified in the country.” If the pathogen was considered relevant, questions on the collection of indicators specifically for that pathogen were then proposed. The questionnaire version for animal virologists and veterinary public health workers included only zoonotic pathogens.

In May 2019, the three versions of the online questionnaire were submitted to 110 officially appointed contact points of the 22 countries of the MLS network. The version focusing on vectors was sent to entomologists, that on human indicators to human virologists and human public health professionals, and that on animal indicators to animal virologists and veterinary services professionals. Several reminders were sent out for completion from June to July, and answers were collected until September 2019. Information collected through the survey administration app was then compiled and analyzed using MS Excel 2016©.

## 3. Results

### 3.1. Selected Indicators

The indicators selected among those identified from the literature are reported in Table 1.

Vectors (mosquitoes for six out of seven selected pathogens, and ticks for CCHFV) were investigated in terms of presence, abundance/density, seasonality, and infection rate.

For humans, two general indicators (population density and population age distribution), and three pathogen-specific indicators regarding disease frequency or occurrence were considered.

The animal-related version of the questionnaire investigated only three zoonotic pathogens: CCHFV, RVFV, and WNV. Questions regarding the collection of two general indicators were asked (animal population density, and animal movements and trade). Furthermore, the pathogen-specific indicators were animal disease occurrence and animal disease seroprevalence.

### 3.2. Questionnaire Responsiveness

Overall, 21 of the 22 MLS network countries responded with at least one completed questionnaire. Countries’ responsiveness was higher in the entomology sector (20/22), followed by the animal (30/44) and human sectors (28/44). In some of the MLS countries, the public health sector is divided on a regional basis, therefore in two cases more than one questionnaire was completed for the same sector. As a result, a total number of 81 questionnaires were collected and analyzed: 20 from entomologists, 31 from animal virologists and the official veterinary sector, and 30 from human virologists and the public health sector. Zoonotic pathogens (CCHFV, RVFV, and WNV) were investigated in 81 questionnaires, while 50 questionnaires concerned non-zoonotic pathogens (CHIKV, DENV, YFV, and ZIKV).

### 3.3. Pathogens Relevance

#### 3.3.1. National Level

From the questionnaire responses, WNV was perceived as the most relevant zoonotic pathogen in the study area, followed by CCHFV and RVFV. Specifically, WNV was relevant for 73% (59/81) of respondents, CCHFV for 63% (51/81), and RVFV for 49% (40/81) (Table 2). Among the non-zoonotic pathogens, DENV was perceived as the most important in the study area as it was considered relevant for 62% (31/50) of the respondents (Table 3). Less relevant non-zoonotic pathogens were CHIKV at 46% (23/50), ZIKV at 40% (20/50), and YFV at 38% (19/50). Percentages of relevance per individual pathogen in the overall study area and within the different sectors are reported below in Table 2 and Table 3.

#### 3.3.2. Regional Level

In line with the aim of implementing early detection strategies in the MLS network area, the regional relevance of the selected pathogens was analyzed (Table 4). To accomplish this, the 22 countries of the study area were grouped in five regions, namely Balkans (Albania, Bosnia & Herzegovina, Kosovo, Montenegro, Republic of North Macedonia, and Serbia), Black Sea (Armenia, Georgia, and Turkey), Middle East (Jordan, Lebanon, and Palestine), North Africa (Algeria, Egypt, Libya, Morocco, and Tunisia), and Sahel (Burkina Faso, Mali, Mauritania, Niger, and Senegal).

Within the regionally grouped data, WNV was the most relevant pathogen for the respondents from Balkans and North Africa; for Black Sea region CCHFV was the most important; in Middle East region DENV was considered relevant, whereas all respondents from the Sahel region considered RVFV as the most important.

### 3.4. Surveillance Systems

To investigate the surveillance systems in place in each sector for the different pathogens, questions about which indicators are routinely collected were asked. Table 5, Table 6 and Table 7 show the level of collection of each indicator for the four most relevant pathogens (WNV, CCHFV, DENV, and RVFV) in the study area.

The most frequently collected indicator in the entomology sector is “vector presence,” regardless of the involved pathogen, while data on “vector infection rate” are rarely gathered (Table 5).

The results from the human sector questionnaires show that population density and population age distribution data are collected in almost all cases (Table 6). To evaluate disease frequency or occurrence, the most frequently collected data are new cases (new notified cases or outbreaks, according to the national case definition, per year) and laboratory cases (number of confirmed laboratory cases, according to national case definition, per year).

Data on animal population are collected in 100% of the cases for cattle, goats, and sheep, and to a lesser extent for equids and camels; these data are rarely collected for wild species. Data on animal movements and trade are frequently collected in terms of import and export, and quite often for pastoralism and transhumance practices. Wild animal information (wildlife migrations) is again gathered very rarely. Data regarding disease occurrence and disease seroprevalence in animals are collected to a different extent depending on the pathogen considered (Table 7).

## 4. Discussion

As stated by Leta et al. [41], arboviral diseases are indeed a global public health threat considering that 215 countries/territories are potentially suitable for the most important arboviral disease vectors (*A. aegypti* and *A. albopictus*) and more than half of these areas are reporting cases of Zika, Dengue fever, Chikungunya, Yellow fever, and/or Rift Valley fever.

The pathogens’ relevance perceived by the respondents of our survey is in accordance with their diffusion in the region. WNV is the most widely distributed arbovirus on the planet [42]; RVFV is relevant mainly for African countries, even if it is suggested that other regions of the MLS network are suitable for potentially competent RVFV vectors and may be considered at risk of introduction through uncontrolled movements of infected animals from infected neighboring countries [43]. CCHFV, clearly acknowledged in the Black Sea and Balkans regions as a priority, is not considered relevant by the respondents from both the Middle East and North Africa, despite its main vector being present in both regions, and virological and serological evidences were highlighted in some of those countries [44]. The results for YFV suggest that it is perceived as the least important pathogen, perhaps in light of the widespread and effective vaccination programs in progress for 50 years [45]. Overall, the relevance ranking of pathogens is the same within the three sectors, demonstrating that shared priorities exist and that there is strong potential for integrated strategies.

According to the information gathered by our survey, the collection of indicators suitable for early warning is in place with a range of different situations in the MLS network countries. At this stage, it would be possible, albeit challenging, to identify opportunities for targeting disease threats upstream (prevention at the source, or via early detection and effective response) in order to support the reduction of occurrence and impact of arboviruses transmission [46].

From the questionnaire results, vector presence predictably resulted as the most collected vector indicator. On the contrary, vector infection rate was the most difficult to gather in case of mosquitos-transmitted pathogens, while for CCHFV (transmitted by ticks) the least collected indicator is vector abundance/density. Therefore, vector species-specific differences should be taken into consideration when interpreting these results. This said, a limitation of the present study is the use of the same indicators regardless of the vector species involved (for example, does data on “vector abundance/density” have the same significance for ticks rather than for mosquitoes?). Moreover, different people and/or different laboratories of the same institution may focus on specific vector species (i.e., mosquitoes or ticks); as a result, addressing our questionnaire to only one of them may have limited the collection of information or biased the results. However, in order to mitigate this risk, we strongly recommended our contact points to share the questionnaire with relevant colleagues when appropriate. Information on vector seasonality is instead less available, although by analyzing the collection of this indicator by country and by pathogen, coherent explanations might be found (e.g., endemicity of the pathogen in the concerned country with the assumption that the vector is abundant throughout the year). Nevertheless, lack of awareness on the importance of some indicators for the monitoring of certain pathogens could also explain their unsuccessful collection.

Data on disease frequency or occurrence in humans (both either suspected cases or lab confirmed) is collected by the majority of the countries involved. On the other hand, less effort is dedicated to seroprevalence studies, despite their potential importance in areas where the disease has been not reported yet.

Interestingly, information on animal population density is collected in all the involved countries for some domestic species, supplying national authorities with important data for animal disease surveillance. However, information regarding wildlife is rarely gathered: surveillance programs in wildlife are needed and desirable, not only in order to investigate the health status of wildlife populations, but also to investigate on potential reservoir of infection and, therefore, to prevent the spillover in animals and humans [47].

A final section in all the questionnaires allowed for the collection of information on indicators relevant to climate and environment (temperature, precipitations, meteorological stations, vegetation, land use, land cover, and soil type). The preliminary analysis of this information shows that this collection should be strengthened, especially in light of the increasing environmental and climate impact on the diffusion of arboviruses.

Collecting data through an online questionnaire proved to be the most convenient and effective solution to quickly gather information from the MLS region. However, this method has some disadvantages: it was not possible to verify the quality (completeness, uniformity, etc.) of data collection in place, nor to investigate individual special cases discovered with the study.

Having assessed that each of the sectors involved in the surveillance was collecting indicators for the most relevant arboviral infections in the MLS region, the next step is to verify if a specific selection of appropriate indicators amongst those presently collected can provide the ability to predict or allow for early warning, especially in terms of a One Health perspective (“integrated early warning”).

The piloting of such a type of integrated approach could help to understand if integrated early warning represents the Achilles’ heel of OH, until more effort will be put into setting up all the requirements needed to operationalize such a system. Among others, the need to increase the performance of the system is crucial in terms of sensibility and specificity through the integration of data from different sources and Institutions. This is only possible with the presence of shared or closely connected information systems, procedures, and indicators.

## 5. Conclusions

A certain grade of collection of surveillance data (indicators) is already in place in the MLS region. Their collection should be strengthened and the gaps on critical indicators addressed (i.e., vector infection rate and wildlife information).

It appears now worthwhile proceeding with further assessments which can help clarify the reliability of the indicators collected and the feasibility of the implementation of an integrated early warning system for arboviral infections. Since some relevant indicators are already being collected, this would not demand for extra resources but, on the contrary, its operationalization could lead to savings. Deep studies in promising countries could help to address the problem and would represent the chance to pilot integrated early warning systems using data already being collected and to promote the collection of critical data not yet gathered.

As stated by Berthe et al.: “One Health is a sound management approach, fully aligned with the definition of ‘health,’ and good practice for its predicament on the use of increasingly scarce resources, therefore improving efficiency and efficacy” [46]. Let us see if this is also the case of early warning for arboviral infections.

## Figures and Tables

**Table 1:** Selected potential indicators for early warning.

Sector	Type of Indicator	Specific Indicators
Vector	Pathogen-specific	Vector presence
		Vector abundance/density
		Vector seasonality
		Vector infection rate
Human	General	Population density
		Population age distribution
	Pathogen-specific	Disease frequency or occurrence—new notified cases/outbreaks (according to National case definition) per year
		Disease frequency or occurrence—number confirmed laboratory cases (according to National case definition) per year
		Disease frequency or occurrence—persons with detected antibodies (sero-prevalence)
Animal	General	Animal population density ^1^
		Animal movements and trade—pastoralism and transhumance
		Animal movements and trade—import and export
		Animal movements and trade—wildlife migrations
	Pathogen-specific ^2^	Animal disease occurrence
		Animal disease seroprevalence

^1^ We investigated the collection of the same indicator in different animal species/group (cattle, goats, sheep, equids, camels, wild ruminants, non-ruminant wild animals, and wild birds). ^2^ Per each pathogen, we investigated the collection of the same indicator in the susceptible animal species: for Crimean-Congo haemorrhagic fever virus (CCHFV), cattle, goats, sheep, camels, wild ruminants, non-ruminant wild animals, and wild birds; for Rift Valley fever virus (RVFV), cattle, goats, sheep, camels, wild ruminants, and non-ruminant wild animals; lastly, for West Nile virus (WNV), equids and wild birds.

**Table 2:** Zoonotic pathogens perceived relevance in the study area and by sectors.

Pathogens	Overall Relevance in the Study Area	Sector Relevance (Vector)	Sector Relevance (Human)	Sector Relevance (Animal)
West Nile virus	59/81 (73%)	18/20 (90%)	22/30 (73%)	18/31 (61%)
Crimean-Congo Haemorrhagic Fever virus	51/81 (63%)	14/20 (70%)	20/30 (67%)	17/31 (55%)
Rift Valley fever virus	40/81 (49%)	11/20 (55%)	13/30 (43%)	16/31 (52%)

**Table 3:** Non-zoonotic pathogens perceived relevance in the study area and by sectors.

Pathogens	Overall Relevance in the Study Area	Sector Relevance (Vector)	Sector Relevance (Human)
Dengue virus	31/50 (62%)	13/20 (65%)	18/30 (60%)
Chikungunya virus	23/50 (46%)	11/20 (55%)	12/30 (40%)
Zika virus	20/53 (40%)	9/20 (45%)	11/30 (37%)
Yellow fever virus	19/53 (38%)	8/20 (40%)	11/30 (37%)

**Table 4:** Regional relevance of the investigated pathogens.

Region	CHIKV	CCHFV	DENV	YFV	RVFV	WNV	ZIKV
Balkans	8/16 (50%)	22/27 (81%)	9/16 (56%)	5/16 (31%)	10/27 (37%)	26/27 (96%)	6/16 (38%)
Black Sea	1/7 (14%)	9/11 (82%)	1/7 (14%)	1/7 (14%)	1/11 (9%)	7/11 (64%)	1/7 (14%)
Middle East	5/6 (83%)	4/11 (36%)	6/6 (100%)	3/6 (50%)	5/11 (45%)	7/11 (64%)	3/6 (50%)
North Africa	5/13 (38%)	6/19 (32%)	8/13 (62%)	6/13 (46%)	11/19 (58%)	16/19 (84%)	6/13 (46%)
Sahel	4/8 (50%)	10/13 (77%)	7/8 (88%)	4/8 (50%)	13/13 (100%)	3/13 (23%)	4/8 (50%)

**Table 5:** Collection of selected indicators for vectors for WNV, CCHFV, Dengue virus (DENV), and RVFV.

Indicators Collected-Vector	WNV	CCHFV	DENV	RVFV
Vector presence	12/18 (67%)	7/14 (50%)	11/13 (85%)	5/11 (45%)
Vector abundance/density	8/18 (44%)	2/14 (14%)	9/13 (69%)	4/11 (36%)
Vector seasonality	8/18 (44%)	4/14 (29%)	10/13 (77%)	3/11 (27%)
Vector infection rate	6/18 (33%)	3/14 (21%)	1/13 (8%)	1/11 (9%)

**Table 6:** Collection of selected indicators for humans for WNV, CCHFV, DENV, and RVFV.

Indicators Collected-Human	General Indicators	WNV	CCHFV	DENV	RVFV
Population density	27/30 (90%)				
Population age distribution	27/30 (90%)				
Disease frequency or occurrence—new notified cases/outbreaks (according to National case definition) per year		20/22 (91%)	18/20 (90%)	14/18 (78%)	10/13 (77%)
Disease frequency or occurrence—number of confirmed laboratory cases (according to National case definition) per year		19/22 (86%)	17/20 (85%)	13/18 (72%)	10/13 (77%)
Disease frequency or occurrence—persons with specific antibodies (seroprevalence)		13/22 (59%)	11/20 (55%)	9/18 (50%)	7/13 (54%)

**Table 7:** Collection of selected indicators for animals for WNV, CCHFV, and RVFV.

Indicators Collected-Animals	General Indicators	WNV	CCHFV	RVFV
Animal population density ^1^	31/31 (100%)			
Animal movements and trade—pastoralism and transhumance	20/31 (65%)			
Animal movements and trade—import and export	30/31 (97%)			
Animal movements and trade—wildlife migrations	5/31 (16%)			
Animal disease occurrence ^2^		14/19 (74%)	8/17 (47%)	11/16 (69%)
Animal disease seroprevalence ^2^		12/19 (63%)	8/17 (47%)	10/16 (63%)

^1^ Animal population information collection involves 100% of the cases for cattle, goats, and sheep, and to a lesser extent for equids and camels, while rarely for wild species. ^2^ These indicators correspond to equids for WNV and cattle, goats, and sheep for CCHFV and RVFV.

## Supplementary Materials

The following are available online at https://www.mdpi.com/2076-2607/8/1/84/s1.

## Appendix A

MediLabSecure Working group: Gloria Nacca and Alessia Ranghiasci (National Centre for Global Health, Istituto Superiore di Sanità, Italy); Barbara Alessandrini, Daria Di Sabatino and Ombretta Pediconi (Istituto Zooprofilattico Sperimentale dell’Abruzzo e del Molise ‘G.Caporale’, Italy); Lobna Gaayeb, Zelie Godin, Ariane Guillot, Vanessa Lagal, Oriane Puechal, and Maud Seguy (Institut Pasteur, France); Jean-Claude Manuguerra and Guillain Mikaty (Institut Pasteur, France); Jovita Fernández-Pinero, Miguel Angel Jiménez-Clavero and Elisa Pérez-Ramírez (Centro de Investigación en Sanidad Animal-Instituto Nacional de Investigación y Tecnología Agraria y Alimentaria, Spain); Marie Picard and Vincent Robert (MIVEGEC unit, Institut de Recherche pour le Développement, Centre National de Recherche Scientifique, Université de Montpellier, France); Guy Hendrickx (Avia-GIS, Belgium).
