# Supplementary material for: Integrated Early Warning Surveillance: Achilles′ Heel of One Health?
Source: Microorganisms. 2020 Jan 8;8(1):84. doi: 10.3390/microorganisms8010084 (PMC7022449; doi:10.3390/microorganisms8010084)
Supplement: Supplementary file 1 [file microorganisms-08-00084-s001.zip › Supplementary_Mat1_QuestionnaireVector.pdf]

# Indicators for Early Warning and Risk Assessment - VECTOR

Contact information

*Required

1. **Email address** *

## MediLabSecure (MLS) One Health Project - Surveillance of Arbovirus infections

## Purpose of the survey

Goal of this survey is to collect information on important indicators for risk assessment and early warning of arboviruses of relevance in the countries of the MLS Network, focusing on IF and HOW such information are collected at country level.

Please, complete each part of the questionnaire in the most accurate way possible.
For any further clarification, feel free to contact us at laura.amato@iss.it

Many thanks in advance for your time and consideration.

The Public Health Work Package of MLS

## Structure of the questionnaire

The present questionnaire focuses on 7 relevant pathogens (namely Chikungunya virus, Crimean-Congo Haemorragic fever virus, Dengue fever virus, Yellow fever virus, Rift Valley fever virus, West Nile virus, Zika virus).

Each sector involved in the surveillance activities of the above pathogens (vector, human, animal) collects data in accordance with respective surveillance priorities. You are kindly asked to fill in this questionnaire that is specifically related to your field of expertise and sector of activity.

Each section is dedicated to one pathogen. At the beginning of each section, a question asking on the relevance of the pathogen for your country lets you provide information on the pathogen or skip to the next one.

Good luck and thank you for your support!

## Vector

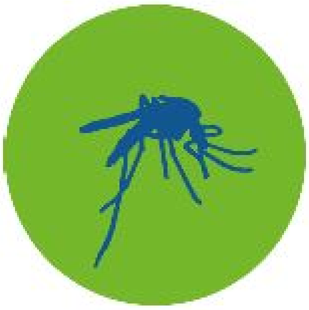

2. **Country** *

3. **Family name** *

4. **Given name** *

5. **Name of your Institution** *

6. **Name of your Laboratory** *

7. **Of which MediLabSecure networks is your laboratory part of?** *

*Tick all that apply.*

- [ ] Human Virology
- [ ] Animal Virology
- [ ] Medical Entomology
- [ ] Human Public Health
- [ ] Animal Public Health
- [ ] Other: ____________

8. **Family name, given name and affiliation of other contributors to the compilation of the survey, if any**

## Chikungunya virus

9. **Is Chikungunya virus a pathogen of relevance for your country? (A relevant pathogen could be an endemic or epidemic pathogen in the country, or an emerging pathogen not yet identified in the country)** *

*Mark only one oval.*

- ( ) YES
- ( ) NO *Skip to question 23.*
- ( ) Other: ____________

## Chikungunya virus

10. **Is your country collecting data on VECTOR PRESENCE and at which level?** *
*Tick all that apply.*

- [ ] NO
- [ ] YES - National aggregated
- [ ] YES - Regional aggregated
- [ ] YES - Local or GPS
- [ ] I don't know
- [ ] Other: ______________________

11. **If yes, where are the collected data on VECTOR PRESENCE stored?**
*Tick all that apply.*

- [ ] Digitalized national database interoperable or integrated with other sectors' databases
- [ ] Digitalized national database
- [ ] Non digitalized national database
- [ ] Local or regional database
- [ ] I don't know
- [ ] Other: ______________________

12. **Is your country collecting data on VECTOR ABUNDANCE/DENSITY and at which level?** *
*Tick all that apply.*

- [ ] NO
- [ ] YES - National aggregated
- [ ] YES - Regional aggregated
- [ ] YES - Local or GPS
- [ ] I don't know
- [ ] Other: ______________________

13. **If yes, which specific data on VECTOR ABUNDANCE/DENSITY is your country collecting?**
*Tick all that apply.*

- [ ] Mosquitoes/ha
- [ ] Mosquitoes/trap
- [ ] Mosquitoes/human
- [ ] Percentage of municipalities with the presence of the vector in a given area
- [ ] I don't know
- [ ] Other: ______________________

14. **If yes, where are the collected data on VECTOR ABUNDANCE/DENSITY stored?**
*Tick all that apply.*

- [ ] Digitalized national database interoperable or integrated with other sectors' databases
- [ ] Digitalized national database
- [ ] Non digitalized national database
- [ ] Local or regional database
- [ ] I don't know
- [ ] Other: ____________________

15. **Is your country collecting data on VECTOR SEASONALITY and at which level?** *
*Tick all that apply.*

- [ ] NO
- [ ] YES - National aggregated
- [ ] YES - Regional aggregated
- [ ] YES - Local or GPS
- [ ] I don't know
- [ ] Other: ____________________

16. **If yes, which specific data on VECTOR SEASONALITY is your country collecting?**
*Tick all that apply.*

- [ ] Weeks of vector activity/year
- [ ] Months of vector activity/year
- [ ] Season of vector activity/year
- [ ] I don't know
- [ ] Other: ____________________

17. **If yes, where are the collected data on VECTOR SEASONALITY stored?**
*Tick all that apply.*

- [ ] Digitalized national database interoperable or integrated with other sectors' databases
- [ ] Digitalized national database
- [ ] Non digitalized national database
- [ ] Local or regional database
- [ ] I don't know
- [ ] Other: ____________________

18. **Is your country collecting data on VECTOR INFECTION RATE and at which level?** *
*Tick all that apply.*

- [ ] NO
- [ ] YES - National aggregated
- [ ] YES - Regional aggregated
- [ ] YES - Local or GPS
- [ ] I don't know
- [ ] Other: ____________________

19. **If yes, which specific data on VECTOR INFECTION RATE is your country collecting?**
*Tick all that apply.*

- [ ] N. positive pools/total tested pools
- [ ] I don't know
- [ ] Other: ____________________

20. **If yes, where are the collected data on VECTOR INFECTION RATE stored?**
*Tick all that apply.*

- [ ] Digitalized national database interoperable or integrated with other sectors' databases
- [ ] Digitalized national database
- [ ] Non digitalized national database
- [ ] Local or regional database
- [ ] I don't know
- [ ] Other: ____________________

21. **Has your office access to any GLOBAL PUBLIC DATASET related to vectors?** *
*Mark only one oval.*

- ( ) Yes
- ( ) No
- ( ) I don't know
- ( ) Other: ____________________

22. **If yes, which one/ones? Are you using it/them for which purpose?**

____________________
____________________
____________________
____________________
____________________

23. **Is your country collecting ANY OTHER RELEVANT INDICATOR not mentioned above? If yes, could you specify?** *

____________________
____________________
____________________
____________________
____________________

# Crimean-Congo Haemorragic fever virus

24. **Is Crimean-Congo Haemorragic fever virus a pathogen of relevance for your country? (A relevant pathogen could be an endemic or epidemic pathogen in the country, or an emerging pathogen not yet identified in the country)** *

*Mark only one oval.*

- YES
- NO *Skip to question 38.*
- Other: ______________________

## Crimean-Congo Haemorragic fever virus

25. **Is your country collecting data on VECTOR PRESENCE and at which level?** *

*Tick all that apply.*

- NO
- YES - National aggregated
- YES - Regional aggregated
- YES - Local or GPS
- I don't know
- Other: ______________________

26. **If yes, where are the collected data on VECTOR PRESENCE stored?**

*Tick all that apply.*

- Digitalized national database interoperable or integrated with other sectors' databases
- Digitalized national database
- Non digitalized national database
- Local or regional database
- I don't know
- Other: ______________________

27. **Is your country collecting data on VECTOR ABUNDANCE/DENSITY and at which level?** *

*Tick all that apply.*

- NO
- YES - National aggregated
- YES - Regional aggregated
- YES - Local or GPS
- I don't know
- Other: ______________________

28. **If yes, which specific data on VECTOR ABUNDANCE/DENSITY is your country collecting?**
*Tick all that apply.*

- [ ] Ticks/ha
- [ ] Ticks/trap
- [ ] Ticks/human or ticks/host
- [ ] Percentage of municipalities with the presence of the vector in a given area
- [ ] I don't know
- [ ] Other: ______________________

29. **If yes, where are the collected data on VECTOR ABUNDANCE/DENSITY stored?**
*Tick all that apply.*

- [ ] Digitalized national database interoperable or integrated with other sectors' databases
- [ ] Digitalized national database
- [ ] Non digitalized national database
- [ ] Local or regional database
- [ ] I don't know
- [ ] Other: ______________________

30. **Is your country collecting data on VECTOR SEASONALITY and at which level?** *
*Tick all that apply.*

- [ ] NO
- [ ] YES - National aggregated
- [ ] YES - Regional aggregated
- [ ] YES - Local or GPS
- [ ] I don't know
- [ ] Other: ______________________

31. **If yes, which specific data on VECTOR SEASONALITY is your country collecting?**
*Tick all that apply.*

- [ ] Weeks of vector activity/year
- [ ] Months of vector activity/year
- [ ] Season of vector activity/year
- [ ] I don't know
- [ ] Other: ______________________

32. **If yes, where are the collected data on VECTOR SEASONALITY stored?**
*Tick all that apply.*

- [ ] Digitalized national database interoperable or integrated with other sectors' databases
- [ ] Digitalized national database
- [ ] Non digitalized national database
- [ ] Local or regional database
- [ ] I don't know
- [ ] Other: ______________________

33. **Is your country collecting data on VECTOR INFECTION RATE and at which level?** *

*Tick all that apply.*

- [ ] NO
- [ ] YES - National aggregated
- [ ] YES - Regional aggregated
- [ ] YES - Local or GPS
- [ ] I don't know
- [ ] Other: ______________________

34. **If yes, which specific data on VECTOR INFECTION RATE is your country collecting?**

*Tick all that apply.*

- [ ] N. positive ticks/total tested ticks
- [ ] I don't know
- [ ] Other: ______________________

35. **If yes, where are the collected data on VECTOR INFECTION RATE stored?**

*Tick all that apply.*

- [ ] Digitalized national database interoperable or integrated with other sectors' databases
- [ ] Digitalized national database
- [ ] Non digitalized national database
- [ ] Local or regional database
- [ ] I don't know
- [ ] Other: ______________________

36. **Has your office access to any GLOBAL PUBLIC DATASET related to vectors?** *

*Mark only one oval.*

- ( ) Yes
- ( ) No
- ( ) I don't know
- ( ) Other: ______________________

37. **If yes, which one/ones? Are you using it/them for which purpose?**

______________________
______________________
______________________
______________________
______________________

38. **Is your country collecting ANY OTHER RELEVANT INDICATOR not mentioned above? If yes, could you specify?** *

_______________
_______________
_______________
_______________
_______________

## Dengue virus

39. **Is Dengue virus a pathogen of relevance for your country? (A relevant pathogen could be an endemic or epidemic pathogen in the country, or an emerging pathogen not yet identified in the country)** *

*Mark only one oval.*

- ( ) YES
- ( ) NO *Skip to question 54.*
- ( ) Other: _______________

## Dengue virus

40. **Did you already provided information on this vector, answering to the questions in the Chikungunya Section?** *

*Mark only one oval.*

- ( ) YES *Skip to question 54.*
- ( ) NO

## Dengue virus

41. **Is your country collecting data on VECTOR PRESENCE and at which level?** *

*Tick all that apply.*

- [ ] NO
- [ ] YES - National aggregated
- [ ] YES - Regional aggregated
- [ ] YES - Local or GPS
- [ ] I don't know
- [ ] Other: _______________

42. **If yes, where are the collected data on VECTOR PRESENCE stored?**

*Tick all that apply.*

- [ ] Digitalized national database interoperable or integrated with other sectors' databases
- [ ] Digitalized national database
- [ ] Non digitalized national database
- [ ] Local or regional database
- [ ] I don't know
- [ ] Other: _______________

43. **Is your country collecting data on VECTOR ABUNDANCE/DENSITY and at which level? ***

*Tick all that apply.*

- [ ] NO
- [ ] YES - National aggregated
- [ ] YES - Regional aggregated
- [ ] YES - Local or GPS
- [ ] I don't know
- [ ] Other: ______________________

44. **If yes, which specific data on VECTOR ABUNDANCE/DENSITY is your country collecting?**

*Tick all that apply.*

- [ ] Mosquitoes/ha
- [ ] Mosquitoes/trap
- [ ] Mosquitoes/human
- [ ] Percentage of municipalities with the presence of the vector in a given area
- [ ] I don't know
- [ ] Other: ______________________

45. **If yes, where are the collected data on VECTOR ABUNDANCE/DENSITY stored?**

*Tick all that apply.*

- [ ] Digitalized national database interoperable or integrated with other sectors' databases
- [ ] Digitalized national database
- [ ] Non digitalized national database
- [ ] Local or regional database
- [ ] I don't know
- [ ] Other: ______________________

46. **Is your country collecting data on VECTOR SEASONALITY and at which level? ***

*Tick all that apply.*

- [ ] NO
- [ ] YES - National aggregated
- [ ] YES - Regional aggregated
- [ ] YES - Local or GPS
- [ ] I don't know
- [ ] Other: ______________________

47. **If yes, which specific data on VECTOR SEASONALITY is your country collecting?**

*Tick all that apply.*

- [ ] Weeks of vector activity/year
- [ ] Months of vector activity/year
- [ ] Season of vector activity/year
- [ ] I don't know
- [ ] Other: ______________________

48. **If yes, where are the collected data on VECTOR SEASONALITY stored?**
*Tick all that apply.*

- [ ] Digitalized national database interoperable or integrated with other sectors' databases
- [ ] Digitalized national database
- [ ] Non digitalized national database
- [ ] Local or regional database
- [ ] I don't know
- [ ] Other: ____________________

49. **Is your country collecting data on VECTOR INFECTION RATE and at which level?** *
*Tick all that apply.*

- [ ] NO
- [ ] YES - National aggregated
- [ ] YES - Regional aggregated
- [ ] YES - Local or GPS
- [ ] I don't know
- [ ] Other: ____________________

50. **If yes, which specific data on VECTOR INFECTION RATE is your country collecting?**
*Tick all that apply.*

- [ ] N. positive pools/total tested pools
- [ ] I don't know
- [ ] Other: ____________________

51. **If yes, where are the collected data on VECTOR INFECTION RATE stored?**
*Tick all that apply.*

- [ ] Digitalized national database interoperable or integrated with other sectors' databases
- [ ] Digitalized national database
- [ ] Non digitalized national database
- [ ] Local or regional database
- [ ] I don't know
- [ ] Other: ____________________

52. **Has your office access to any GLOBAL PUBLIC DATASET related to vectors?** *
*Mark only one oval.*

- ( ) Yes
- ( ) No
- ( ) I don't know
- ( ) Other: ____________________

53. **If yes, which one/ones? Are you using it/them for which purpose?**

54. **Is your country collecting ANY OTHER RELEVANT INDICATOR not mentioned above? If yes, could you specify?** *

# Yellow Fever virus

55. **Is Yellow Fever virus a pathogen of relevance for your country? (A relevant pathogen could be an endemic or epidemic pathogen in the country, or an emerging pathogen not yet identified in the country)** *

*Mark only one oval.*

- YES
- NO *Skip to question 70.*
- Other: ____

# Yellow Fever virus

56. **Did you already provided information on this vector, answering to the questions in the Chikungunya or Dengue Sections?** *

*Mark only one oval.*

- YES *Skip to question 70.*
- NO

# Yellow Fever virus

57. **Is your country collecting data on VECTOR PRESENCE and at which level?** *

*Tick all that apply.*

- [ ] NO
- [ ] YES - National aggregated
- [ ] YES - Regional aggregated
- [ ] YES - Local or GPS
- [ ] I don't know
- [ ] Other: ____

58. **If yes, where are the collected data on VECTOR PRESENCE stored?**
*Tick all that apply.*

- [ ] Digitalized national database interoperable or integrated with other sectors' databases
- [ ] Digitalized national database
- [ ] Non digitalized national database
- [ ] Local or regional database
- [ ] I don't know
- [ ] Other: ____________________

59. **Is your country collecting data on VECTOR ABUNDANCE/DENSITY and at which level?** *
*Tick all that apply.*

- [ ] NO
- [ ] YES - National aggregated
- [ ] YES - Regional aggregated
- [ ] YES - Local or GPS
- [ ] I don't know
- [ ] Other: ____________________

60. **If yes, which specific data on VECTOR ABUNDANCE/DENSITY is your country collecting?**
*Tick all that apply.*

- [ ] Mosquitoes/ha
- [ ] Mosquitoes/trap
- [ ] Mosquitoes/human
- [ ] Percentage of municipalities with the presence of the vector in a given area
- [ ] I don't know
- [ ] Other: ____________________

61. **If yes, where are the collected data on VECTOR ABUNDANCE/DENSITY stored?**
*Tick all that apply.*

- [ ] Digitalized national database interoperable or integrated with other sectors' databases
- [ ] Digitalized national database
- [ ] Non digitalized national database
- [ ] Local or regional database
- [ ] I don't know
- [ ] Other: ____________________

62. **Is your country collecting data on VECTOR SEASONALITY and at which level?** *

*Tick all that apply.*

- [ ] NO
- [ ] YES - National aggregated
- [ ] YES - Regional aggregated
- [ ] YES - Local or GPS
- [ ] I don't know
- [ ] Other: ______________________

63. **If yes, which specific data on VECTOR SEASONALITY is your country collecting?**

*Tick all that apply.*

- [ ] Weeks of vector activity/year
- [ ] Months of vector activity/year
- [ ] Season of vector activity/year
- [ ] I don't know
- [ ] Other: ______________________

64. **If yes, where are the collected data on VECTOR SEASONALITY stored?**

*Tick all that apply.*

- [ ] Digitalized national database interoperable or integrated with other sectors' databases
- [ ] Digitalized national database
- [ ] Non digitalized national database
- [ ] Local or regional database
- [ ] I don't know
- [ ] Other: ______________________

65. **Is your country collecting data on VECTOR INFECTION RATE and at which level?** *

*Tick all that apply.*

- [ ] NO
- [ ] YES - National aggregated
- [ ] YES - Regional aggregated
- [ ] YES - Local or GPS
- [ ] I don't know
- [ ] Other: ______________________

66. **If yes, which specific data on VECTOR INFECTION RATE is your country collecting?**

*Tick all that apply.*

- [ ] N. positive pools/total tested pools
- [ ] I don't know
- [ ] Other: ______________________

67. **If yes, where are the collected data on VECTOR INFECTION RATE stored?**
*Tick all that apply.*

- [ ] Digitalized national database interoperable or integrated with other sectors' databases
- [ ] Digitalized national database
- [ ] Non digitalized national database
- [ ] Local or regional database
- [ ] I don't know
- [ ] Other: ______________________

68. **Has your office access to any GLOBAL PUBLIC DATASET related to vectors?** *
*Mark only one oval.*

- ( ) Yes
- ( ) No
- ( ) I don't know
- ( ) Other: ______________________

69. **If yes, which one/ones? Are you using it/them for which purpose?**

______________________
______________________
______________________
______________________
______________________

70. **Is your country collecting ANY OTHER RELEVANT INDICATOR not mentioned above? If yes, could you specify?** *

______________________
______________________
______________________
______________________
______________________

## Rift Valley Fever virus

71. **Is Rift Valley Fever virus a pathogen of relevance for your country? (A relevant pathogen could be an endemic or epidemic pathogen in the country, or an emerging pathogen not yet identified in the country)** *
*Mark only one oval.*

- ( ) YES
- ( ) NO *Skip to question 85.*
- ( ) Other: ______________________

## Rift Valley Fever virus

72. **Is your country collecting data on VECTOR PRESENCE and at which level?** *
*Tick all that apply.*

- [ ] NO
- [ ] YES - National aggregated
- [ ] YES - Regional aggregated
- [ ] YES - Local or GPS
- [ ] I don't know
- [ ] Other: ______________________________

73. **If yes, where are the collected data on VECTOR PRESENCE stored?**
*Tick all that apply.*

- [ ] Digitalized national database interoperable or integrated with other sectors’ databases
- [ ] Digitalized national database
- [ ] Non digitalized national database
- [ ] Local or regional database
- [ ] I don't know
- [ ] Other: ______________________________

74. **Is your country collecting data on VECTOR ABUNDANCE/DENSITY and at which level?** *
*Tick all that apply.*

- [ ] NO
- [ ] YES - National aggregated
- [ ] YES - Regional aggregated
- [ ] YES - Local or GPS
- [ ] I don't know
- [ ] Other: ______________________________

75. **If yes, which specific data on VECTOR ABUNDANCE/DENSITY is your country collecting?**
*Tick all that apply.*

- [ ] Mosquitoes/ha
- [ ] Mosquitoes/trap
- [ ] Mosquitoes/human
- [ ] Percentage of municipalities with the presence of the vector in a given area
- [ ] I don't know
- [ ] Other: ______________________________

76. **If yes, where are the collected data on VECTOR ABUNDANCE/DENSITY stored?**
*Tick all that apply.*

- [ ] Digitalized national database interoperable or integrated with other sectors' databases
- [ ] Digitalized national database
- [ ] Non digitalized national database
- [ ] Local or regional database
- [ ] I don't know
- [ ] Other: ______________________

77. **Is your country collecting data on VECTOR SEASONALITY and at which level?** *
*Tick all that apply.*

- [ ] NO
- [ ] YES - National aggregated
- [ ] YES - Regional aggregated
- [ ] YES - Local or GPS
- [ ] I don't know
- [ ] Other: ______________________

78. **If yes, which specific data on VECTOR SEASONALITY is your country collecting?**
*Tick all that apply.*

- [ ] Weeks of vector activity/year
- [ ] Months of vector activity/year
- [ ] Season of vector activity/year
- [ ] I don't know
- [ ] Other: ______________________

79. **If yes, where are the collected data on VECTOR SEASONALITY stored?**
*Tick all that apply.*

- [ ] Digitalized national database interoperable or integrated with other sectors' databases
- [ ] Digitalized national database
- [ ] Non digitalized national database
- [ ] Local or regional database
- [ ] I don't know
- [ ] Other: ______________________

80. **Is your country collecting data on VECTOR INFECTION RATE and at which level?** *
*Tick all that apply.*

- [ ] NO
- [ ] YES - National aggregated
- [ ] YES - Regional aggregated
- [ ] YES - Local or GPS
- [ ] I don't know
- [ ] Other: ______________________

81. **If yes, which specific data on VECTOR INFECTION RATE is your country collecting?**
*Tick all that apply.*

- [ ] N. positive pools/total tested pools
- [ ] I don't know
- [ ] Other: ____________________

82. **If yes, where are the collected data on VECTOR INFECTION RATE stored?**
*Tick all that apply.*

- [ ] Digitalized national database interoperable or integrated with other sectors' databases
- [ ] Digitalized national database
- [ ] Non digitalized national database
- [ ] Local or regional database
- [ ] I don't know
- [ ] Other: ____________________

83. **Has your office access to any GLOBAL PUBLIC DATASET related to vectors?** *
*Mark only one oval.*

- ( ) Yes
- ( ) No
- ( ) I don't know
- ( ) Other: ____________________

84. **If yes, which one/ones? Are you using it/them for which purpose?**

85. **Is your country collecting ANY OTHER RELEVANT INDICATOR not mentioned above? If yes, could you specify?** *

# West Nile virus

86. **Is West Nile Fever virus a pathogen of relevance for your country? (A relevant pathogen could be an endemic or epidemic pathogen in the country, or an emerging pathogen not yet identified in the country)** *

*Mark only one oval.*

- YES
- NO *Skip to question 100.*
- Other: ______________________

## West Nile virus

87. **Is your country collecting data on VECTOR PRESENCE and at which level?** *

*Tick all that apply.*

- [ ] NO
- [ ] YES - National aggregated
- [ ] YES - Regional aggregated
- [ ] YES - Local or GPS
- [ ] I don't know
- [ ] Other: ______________________

88. **If yes, where are the collected data on VECTOR PRESENCE stored?**

*Tick all that apply.*

- [ ] Digitalized national database interoperable or integrated with other sectors' databases
- [ ] Digitalized national database
- [ ] Non digitalized national database
- [ ] Local or regional database
- [ ] I don't know
- [ ] Other: ______________________

89. **Is your country collecting data on VECTOR ABUNDANCE/DENSITY and at which level?** *

*Tick all that apply.*

- [ ] NO
- [ ] YES - National aggregated
- [ ] YES - Regional aggregated
- [ ] YES - Local or GPS
- [ ] I don't know
- [ ] Other: ______________________

90. **If yes, which specific data on VECTOR ABUNDANCE/DENSITY is your country collecting?**
*Tick all that apply.*

- [ ] Mosquitoes/ha
- [ ] Mosquitoes/trap
- [ ] Mosquitoes/human
- [ ] Percentage of municipalities with the presence of the vector in a given area
- [ ] I don't know
- [ ] Other: ____________________

91. **If yes, where are the collected data on VECTOR ABUNDANCE/DENSITY stored?**
*Tick all that apply.*

- [ ] Digitalized national database interoperable or integrated with other sectors' databases
- [ ] Digitalized national database
- [ ] Non digitalized national database
- [ ] Local or regional database
- [ ] I don't know
- [ ] Other: ____________________

92. **Is your country collecting data on VECTOR SEASONALITY and at which level?** *
*Tick all that apply.*

- [ ] NO
- [ ] YES - National aggregated
- [ ] YES - Regional aggregated
- [ ] YES - Local or GPS
- [ ] I don't know
- [ ] Other: ____________________

93. **If yes, which specific data on VECTOR SEASONALITY is your country collecting?**
*Tick all that apply.*

- [ ] Weeks of vector activity/year
- [ ] Months of vector activity/year
- [ ] Season of vector activity/year
- [ ] I don't know
- [ ] Other: ____________________

94. **If yes, where are the collected data on VECTOR SEASONALITY stored?**
*Tick all that apply.*

- [ ] Digitalized national database interoperable or integrated with other sectors' databases
- [ ] Digitalized national database
- [ ] Non digitalized national database
- [ ] Local or regional database
- [ ] I don't know
- [ ] Other: ____________________

95. **Is your country collecting data on VECTOR INFECTION RATE and at which level?** *
*Tick all that apply.*

- [ ] NO
- [ ] YES - National aggregated
- [ ] YES - Regional aggregated
- [ ] YES - Local or GPS
- [ ] I don't know
- [ ] Other: ____________________

96. **If yes, which specific data on VECTOR INFECTION RATE is your country collecting?**
*Tick all that apply.*

- [ ] N. positive pools/total tested pools
- [ ] I don't know
- [ ] Other: ____________________

97. **If yes, where are the collected data on VECTOR INFECTION RATE stored?**
*Tick all that apply.*

- [ ] Digitalized national database interoperable or integrated with other sectors' databases
- [ ] Digitalized national database
- [ ] Non digitalized national database
- [ ] Local or regional database
- [ ] I don't know
- [ ] Other: ____________________

98. **Has your office access to any GLOBAL PUBLIC DATASET related to vectors?** *
*Mark only one oval.*

- ( ) Yes
- ( ) No
- ( ) I don't know
- ( ) Other: ____________________

99. **If yes, which one/ones? Are you using it/them for which purpose?**

____________________
____________________
____________________
____________________
____________________

100. **Is your country collecting ANY OTHER RELEVANT INDICATOR not mentioned above? If yes, could you specify? ***

## Zika virus

101. **Is Zika virus a pathogen of relevance for your country? (A relevant pathogen could be an endemic or epidemic pathogen in the country, or an emerging pathogen not yet identified in the country) ***

*Mark only one oval.*

- YES
- NO *Skip to question 116.*
- Other:

## Zika virus

102. **Did you already provided information on this vector, answering to the questions in the Chikungunya or Dengue or Yellow Fever Sections? ***

*Mark only one oval.*

- YES *Skip to question 116.*
- NO

## Zika virus

103. **Is your country collecting data on VECTOR PRESENCE and at which level? ***

*Tick all that apply.*

- NO
- YES - National aggregated
- YES - Regional aggregated
- YES - Local or GPS
- I don't know
- Other:

104. **If yes, where are the collected data on VECTOR PRESENCE stored?**

*Tick all that apply.*

- Digitalized national database interoperable or integrated with other sectors' databases
- Digitalized national database
- Non digitalized national database
- Local or regional database
- I don't know
- Other:

105. **Is your country collecting data on VECTOR ABUNDANCE/DENSITY and at which level? ***
*Tick all that apply.*

- [ ] NO
- [ ] YES - National aggregated
- [ ] YES - Regional aggregated
- [ ] YES - Local or GPS
- [ ] I don't know
- [ ] Other: ____________________

106. **If yes, which specific data on VECTOR ABUNDANCE/DENSITY is your country collecting?**
*Tick all that apply.*

- [ ] Mosquitoes/ha
- [ ] Mosquitoes/trap
- [ ] Mosquitoes/human
- [ ] Percentage of municipalities with the presence of the vector in a given area
- [ ] I don't know
- [ ] Other: ____________________

107. **If yes, where are the collected data on VECTOR ABUNDANCE/DENSITY stored?**
*Tick all that apply.*

- [ ] Digitalized national database interoperable or integrated with other sectors' databases
- [ ] Digitalized national database
- [ ] Non digitalized national database
- [ ] Local or regional database
- [ ] I don't know
- [ ] Other: ____________________

108. **Is your country collecting data on VECTOR SEASONALITY and at which level? ***
*Tick all that apply.*

- [ ] NO
- [ ] YES - National aggregated
- [ ] YES - Regional aggregated
- [ ] YES - Local or GPS
- [ ] I don't know
- [ ] Other: ____________________

109. **If yes, which specific data on VECTOR SEASONALITY is your country collecting?**
*Tick all that apply.*

- [ ] Months of vector activity/year
- [ ] Weeks of vector activity/year
- [ ] Season of vector activity/year
- [ ] I don't know
- [ ] Other: ____________________

110. **If yes, where are the collected data on VECTOR SEASONALITY stored?**
*Tick all that apply.*

- [ ] Digitalized national database interoperable or integrated with other sectors' databases
- [ ] Digitalized national database
- [ ] Non digitalized national database
- [ ] Local or regional database
- [ ] I don't know
- [ ] Other: ____________________

111. **Is your country collecting data on VECTOR INFECTION RATE and at which level?** *
*Tick all that apply.*

- [ ] NO
- [ ] YES - National aggregated
- [ ] YES - Regional aggregated
- [ ] YES - Local or GPS
- [ ] I don't know
- [ ] Other: ____________________

112. **If yes, which specific data on VECTOR INFECTION RATE is your country collecting?**
*Tick all that apply.*

- [ ] N. positive pools/total tested pools
- [ ] I don't know
- [ ] Other: ____________________

113. **If yes, where are the collected data on VECTOR INFECTION RATE stored?**
*Tick all that apply.*

- [ ] Digitalized national database interoperable or integrated with other sectors' databases
- [ ] Digitalized national database
- [ ] Non digitalized national database
- [ ] Local or regional database
- [ ] I don't know
- [ ] Other: ____________________

114. **Has your office access to any GLOBAL PUBLIC DATASET related to vectors?** *
*Mark only one oval.*

- ( ) Yes
- ( ) No
- ( ) I don't know
- ( ) Other: ____________________

115. **If yes, which one/ones? Are you using it/them for which purpose?**

116. **Is your country collecting ANY OTHER RELEVANT INDICATOR not mentioned above? If yes, could you specify?** *

# Climate & Environment

This section is collecting information on indicators regarding Climate & Environment regardless of the pathogens

# Climate & Environment

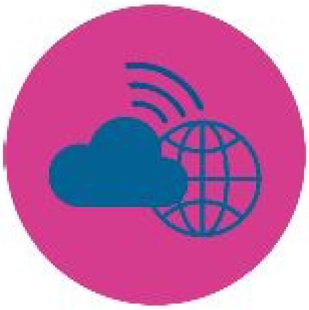

117. **Is your country collecting data on TEMPERATURE and at which level?** *

*Tick all that apply.*

- [ ] NO
- [ ] YES - National aggregated
- [ ] YES - Regional aggregated
- [ ] YES - Local or GPS
- [ ] I don't know
- [ ] Other:

118. **If yes, which specific data on TEMPERATURE is your country collecting?**

*Tick all that apply.*

- [ ] Maximum temperature
- [ ] Annual average of mean temperature
- [ ] I don't know
- [ ] Other:

119. **If yes, where are the collected data on TEMPERATURE stored?**
*Tick all that apply.*

- [ ] Digitalized national database interoperable or integrated with other sectors’ databases
- [ ] Digitalized national database
- [ ] Non digitalized national database
- [ ] Local or regional database
- [ ] I don't know
- [ ] Other: ____________________

120. **Is your country collecting data on PRECIPITATIONS and at which level?** *
*Tick all that apply.*

- [ ] NO
- [ ] YES - National aggregated
- [ ] YES - Regional aggregated
- [ ] YES - Local or GPS
- [ ] I don't know
- [ ] Other: ____________________

121. **If yes, which specific data on PRECIPITATIONS is your country collecting?**
*Tick all that apply.*

- [ ] Monthly precipitation
- [ ] Weekly precipitation
- [ ] I don't know
- [ ] Other: ____________________

122. **If yes, where are the collected data on PRECIPITATIONS stored?**
*Tick all that apply.*

- [ ] Digitalized national database interoperable or integrated with other sectors’ databases
- [ ] Digitalized national database
- [ ] Non digitalized national database
- [ ] Local or regional database
- [ ] I don't know
- [ ] Other: ____________________

123. **Where are the METEOROLOGICAL STATIONS of your country? Please specify place and region. If you don't know, please answer "I don't know".** *

____________________
____________________
____________________
____________________
____________________

124. **Which variables are routinely recorded in your meteorological stations?**
*Tick all that apply.*

- [ ] Temperature
- [ ] Precipitations
- [ ] I don't know
- [ ] Other: ______________________

125. **Is your country collecting data on VEGETATION and at which level?** *
*Tick all that apply.*

- [ ] NO
- [ ] YES - National aggregated
- [ ] YES - Regional aggregated
- [ ] YES - Local or GPS
- [ ] I don't know
- [ ] Other: ______________________

126. **If yes, which specific data on VEGETATION is your country collecting?**

______________________

127. **If yes, where are the collected data on VEGETATION stored?**
*Tick all that apply.*

- [ ] Digitalized national database interoperable or integrated with other sectors' databases
- [ ] Digitalized national database
- [ ] Non digitalized national database
- [ ] Local or regional database
- [ ] I don't know
- [ ] Other: ______________________

128. **Is your country collecting data on LAND USE and at which level?** *
*Tick all that apply.*

- [ ] NO
- [ ] YES - National aggregated
- [ ] YES - Regional aggregated
- [ ] YES - Local or GPS
- [ ] I don't know
- [ ] Other: ______________________

129. **If yes, which specific data on LAND USE is your country collecting?**
*Tick all that apply.*

- [ ] Forest, mixed vegetation, cropland or urban land uses
- [ ] I don't know
- [ ] Other: ______________________

130. **If yes, where are the collected data on LAND USE stored?**
*Tick all that apply.*

- [ ] Digitalized national database interoperable or integrated with other sectors' databases
- [ ] Digitalized national database
- [ ] Non digitalized national database
- [ ] Local or regional database
- [ ] I don't know
- [ ] Other: ______

131. **Is your country collecting data on LAND COVER and at which level?** *
*Tick all that apply.*

- [ ] NO
- [ ] YES - National aggregated
- [ ] YES - Regional aggregated
- [ ] YES - Local or GPS
- [ ] I don't know
- [ ] Other: ______

132. **If yes, which specific data on LAND COVER is your country collecting?**
*Tick all that apply.*

- [ ] Artificial, cultivated, herbaceous cover, tree cover, mosaic and water
- [ ] I don't know
- [ ] Other: ______

133. **If yes, where are the collected data on LAND COVER stored?**
*Tick all that apply.*

- [ ] Digitalized national database interoperable or integrated with other sectors' databases
- [ ] Digitalized national database
- [ ] Non digitalized national database
- [ ] Local or regional database
- [ ] I don't know
- [ ] Other: ______

134. **Is your country collecting data on SOIL TYPE and at which level?** *
*Tick all that apply.*

- [ ] NO
- [ ] YES - National aggregated
- [ ] YES - Regional aggregated
- [ ] YES - Local or GPS
- [ ] I don't know
- [ ] Other: ______

135. **If yes, which specific data on SOIL TYPE is your country collecting?**

136. **If yes, where are the collected data on SOIL TYPE stored?**
*Tick all that apply.*

- [ ] Digitalized national database interoperable or integrated with other sectors' databases
- [ ] Digitalized national database
- [ ] Non digitalized national database
- [ ] Local or regional database
- [ ] I don't know
- [ ] Other:

137. **The above mentioned indicators are collected by which INSTITUTION / INSTITUTIONS? If you don't know, please answer "I don't know".** *

138. **Has your office access to any GLOBAL PUBLIC DATASET related to Climate & Environment?** *
*Mark only one oval.*

- Yes
- No
- I don't know
- Other:

139. **If yes, which one/ones? Are you using it/them for which purpose?**

140. **Is your country collecting ANY OTHER RELEVANT INDICATOR not mentioned above? If yes, could you specify?** *

## Conclusions

141. **Please feel free to put any comment, suggestion or remark here below**

## Thank you very much for your time and consideration!

## MediLabSecure 2 is a project funded by the EC DEVCO (IFS/2018/402-247)

CBRN Centres of Excellence
An initiative of the European Union

## Please be kindly informed that the information you have provided with this questionnaire will be shared and disseminated only in aggregated form with the information provided by all the other MLS Countries involved in this survey.

☐ Send me a copy of my responses.

Powered by
Google Forms
