# Supplementary material for: Integrated Early Warning Surveillance: Achilles′ Heel of One Health?
Source: Microorganisms. 2020 Jan 8;8(1):84. doi: 10.3390/microorganisms8010084 (PMC7022449; doi:10.3390/microorganisms8010084)
Supplement: Supplementary file 1 [file microorganisms-08-00084-s001.zip › Supplementary_Mat2_QuestionnaireHuman.pdf]

Indicators for Early Warning and Risk Assessment - HUMAN

Contact information

*Required

1. **Email address** *

MediLabSecure (MLS) One Health Project - Surveillance of Arbovirus infections

Purpose of the survey

Goal of this survey is to collect information on important indicators for risk assessment and early warning of arboviruses of relevance in the countries of the MLS Network, focusing on IF and HOW such information are collected at country level.

Please, complete each part of the questionnaire in the most accurate way possible.
For any further clarification, feel free to contact us at laura.amato@iss.it

Many thanks in advance for your time and consideration.

The Public Health Work Package of MLS

Structure of the questionnaire

The present questionnaire focuses on 7 relevant pathogens (namely Chikungunya virus, Crimean-Congo Haemorragic fever virus, Dengue fever virus, Yellow fever virus, Rift Valley fever virus, West Nile virus, Zika virus).

Each sector involved in the surveillance activities of the above pathogens (vector, human, animal) collects data in accordance with respective surveillance priorities. You are kindly asked to fill in this questionnaire that is specifically related to your field of expertise and sector of activity.

Each section is dedicated to one pathogen. At the beginning of each section, a question asking on the relevance of the pathogen for your country lets you provide information on the pathogen or skip to the next one.

Good luck and thank you for your support!

Human

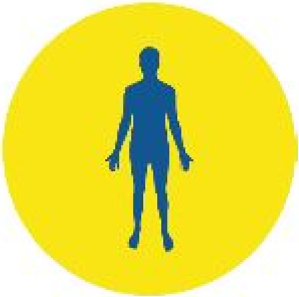

2. **Country** *

3. **Family name** *

4. **Given name** *

5. **Name of your Institution** *

6. **Name of your Laboratory** *

7. **Of which MediLabSecure networks is your laboratory part of?** *
Tick all that apply.

- [ ] Human Virology
- [ ] Animal Virology
- [ ] Medical Entomology
- [ ] Human Public Health
- [ ] Animal Public Health
- [ ] Other: ____________

8. **Family name, given name and affiliation of other contributors to the compilation of the survey, if any**

Public Health - General Section

9. **Is your country collecting data on POPULATION DENSITY and at which level?** *
Tick all that apply.

- [ ] NO
- [ ] YES - National aggregated
- [ ] YES - Regional aggregated
- [ ] YES - Local or municipal
- [ ] I don't know
- [ ] Other: ____________________

10. **If yes, where are the collected data on POPULATION DENSITY stored?**
Tick all that apply.

- [ ] Digitalized national database interoperable or integrated with other sectors' databases
- [ ] Digitalized national database
- [ ] Non digitalized national database
- [ ] Local or regional database
- [ ] I don't know
- [ ] Other: ____________________

11. **Is your country collecting data on POPULATION AGE DISTRIBUTION and at which level?** *
Tick all that apply.

- [ ] NO
- [ ] YES - National aggregated
- [ ] YES - Regional aggregated
- [ ] YES - Local or municipal
- [ ] I don't know
- [ ] Other: ____________________

12. **If yes, where are the collected data on POPULATION AGE DISTRIBUTION stored?**
Tick all that apply.

- [ ] Digitalized national database interoperable or integrated with other sectors' databases
- [ ] Digitalized national database
- [ ] Non digitalized national database
- [ ] Local or regional database
- [ ] I don't know
- [ ] Other: ____________________

13. **Has your office access to any GLOBAL PUBLIC DATASET related to humans?** *
Mark only one oval.

- ( ) Yes
- ( ) No
- ( ) I don't know
- ( ) Other: ____________________

14. **If yes, which one/ones? Are you using it/them for which purpose?**

Chikungunya virus

15. **Is Chikungunya virus a pathogen of relevance for your country? (A relevant pathogen could be an endemic or epidemic pathogen in the country, or an emerging pathogen not yet identified in the country)** *

Mark only one oval.

- YES
- NO *Skip to question 20.*
- Other: ____________

Chikungunya virus

16. **Is your country collecting data on DISEASE FREQUENCY or OCCURRENCE and at which level?** *

Tick all that apply.

	No	YES (national aggregated)	YES (regional aggregated)	YES (local or GPS)	Other	I don't know
New notified cases / outbreaks (according to National case definition) per year	☐	☐	☐	☐	☐	☐
Number confirmed laboratory cases (according to National case definition) per year	☐	☐	☐	☐	☐	☐
Persons with detected antibodies / tested persons (SERO-PREVALENCE)	☐	☐	☐	☐	☐	☐

17. **If you selected "other" in the previous question, please specify here below**

18. **If yes, where are the collected data stored?**
Tick all that apply.

	Digitalized national database interoperable or integrated with other sectors' databases	Digitalized national database	Non digitalized national database	Local or regional database	Other	I don't know	Non applicable
New notified cases / outbreaks (according to National case definition) per year	☐	☐	☐	☐	☐	☐	☐
Number confirmed laboratory cases (according to National case definition) per year	☐	☐	☐	☐	☐	☐	☐
Persons with detected antibodies / tested persons (SERO-PREVALENCE)	☐	☐	☐	☐	☐	☐	☐

19. **If you selected "other" in the previous question, please specify here below**

____

20. **Is your country collecting ANY OTHER RELEVANT INDICATOR not mentioned above? If yes, could you specify?** *

____
____
____
____
____

Crimean-Congo Haemorragic fever virus

21. **Is Crimean-Congo Haemorragic fever virus a pathogen of relevance for your country? (A relevant pathogen could be an endemic or epidemic pathogen in the country, or an emerging pathogen not yet identified in the country)** *
Mark only one oval.

- ◯ YES
- ◯ NO *Skip to question 26.*
- ◯ Other: ______________________________

Crimean-Congo Haemorragic fever virus

22. **Is your country collecting data on DISEASE FREQUENCY or OCCURRENCE and at which level?** *
Tick all that apply.

	No	YES (national aggregated)	YES (regional aggregated)	YES (local or GPS)	Other	I don't know
New notified cases / outbreaks (according to National case definition) per year	☐	☐	☐	☐	☐	☐
Number confirmed laboratory cases (according to National case definition) per year	☐	☐	☐	☐	☐	☐
Persons with detected antibodies / tested persons (SERO-PREVALENCE)	☐	☐	☐	☐	☐	☐

23. **If you selected "other" in the previous question, please specify here below**

____

24. **If yes, where are the collected data stored?**
Tick all that apply.

	Digitalized national database interoperable or integrated with other sectors' databases	Digitalized national database	Non digitalized national database	Local or regional database	Other	I don't know	Non applicable
New notified cases / outbreaks (according to National case definition) per year	☐	☐	☐	☐	☐	☐	☐
Number confirmed laboratory cases (according to National case definition) per year	☐	☐	☐	☐	☐	☐	☐
Persons with detected antibodies / tested persons (SERO-PREVALENCE)	☐	☐	☐	☐	☐	☐	☐

25. **If you selected "other" in the previous question, please specify here below**

____

26. **Is your country collecting ANY OTHER RELEVANT INDICATOR not mentioned above? If yes, could you specify?** *

Dengue virus

27. **Is Dengue virus a pathogen of relevance for your country? (A relevant pathogen could be an endemic or epidemic pathogen in the country, or an emerging pathogen not yet identified in the country)** *

Mark only one oval.

- YES
- NO *Skip to question 37.*
- Other:

Dengue virus

28. **Is your country collecting data on DISEASE FREQUENCY or OCCURRENCE and at which level?** *

Tick all that apply.

	No	YES (national aggregated)	YES (regional aggregated)	YES (local or GPS)	Other	I don't know
New notified cases / outbreaks (according to National case definition) per year	☐	☐	☐	☐	☐	☐
Number confirmed laboratory cases (according to National case definition) per year	☐	☐	☐	☐	☐	☐
Persons with detected antibodies / tested persons (SERO-PREVALENCE)	☐	☐	☐	☐	☐	☐

29. **If you selected "other" in the previous question, please specify here below**

30. **If yes, where are the collected data stored?**
Tick all that apply.

	Digitalized national database interoperable or integrated with other sectors' databases	Digitalized national database	Non digitalized national database	Local or regional database	Other	I don't know	Non applicable
New notified cases / outbreaks (according to National case definition) per year	☐	☐	☐	☐	☐	☐	☐
Number confirmed laboratory cases (according to National case definition) per year	☐	☐	☐	☐	☐	☐	☐
Persons with detected antibodies / tested persons (SERO-PREVALENCE)	☐	☐	☐	☐	☐	☐	☐

31. **If you selected "other" in the previous question, please specify here below**

____

32. **Is your country performing ROUTINELY VACCINATION FOR DENGUE?** *
Tick all that apply.

- ☐ YES
- ☐ NOT AT ALL
- ☐ NOT ROUTINELY / OTHER
- ☐ I DON'T KNOW

33. **If you selected "NOT ROUTINELY / OTHER" in the previous question, could you please specify here below?**

____

34. **Is your country following any specific policy / guidelines regarding the vaccination? Could you please specify or report here below the link?**

____
____
____
____
____

35. **If "YES" or "NOT ROUTINELY / OTHER", is your country collecting data on number of vaccinated per year or vaccination coverage, and at which level?**

Tick all that apply.

- [ ] NO
- [ ] YES - National aggregated
- [ ] YES - Regional aggregated
- [ ] YES - Local or municipal
- [ ] I don't know
- [ ] Other: ______________________

36. **If "YES" or "NOT ROUTINELY / OTHER", where are the collected data on VACCINATION stored?**

Tick all that apply.

- [ ] Digitalized national database interoperable or integrated with other sectors' databases
- [ ] Digitalized national database
- [ ] Non digitalized national database
- [ ] Local or regional database
- [ ] I don't know
- [ ] Other: ______________________

37. **Is your country collecting ANY OTHER RELEVANT INDICATOR not mentioned above? If yes, could you specify?** *

____

____

____

____

____

Yellow Fever virus

38. **Is Yellow Fever virus a pathogen of relevance for your country? (A relevant pathogen could be an endemic or epidemic pathogen in the country, or an emerging pathogen not yet identified in the country)** *

Mark only one oval.

- ( ) YES
- ( ) NO *Skip to question 47.*
- ( ) Other: ______________________

Yellow fever virus

39. **Is your country collecting data on DISEASE FREQUENCY or OCCURRENCE and at which level?** *

Tick all that apply.

	No	YES (national aggregated)	YES (regional aggregated)	YES (local or GPS)	Other	I don't know
New notified cases / outbreaks (according to National case definition) per year	☐	☐	☐	☐	☐	☐
Number confirmed laboratory cases (according to National case definition) per year	☐	☐	☐	☐	☐	☐
Persons with detected antibodies / tested persons (SERO-PREVALENCE)	☐	☐	☐	☐	☐	☐

40. **If you selected "other" in the previous question, please specify here below**

____

41. **If yes, where are the collected data stored?**

Tick all that apply.

	Digitalized national database interoperable or integrated with other sectors' databases	Digitalized national database	Non digitalized national database	Local or regional database	Other	I don't know	Non applicable
New notified cases / outbreaks (according to National case definition) per year	☐	☐	☐	☐	☐	☐	☐
Number confirmed laboratory cases (according to National case definition) per year	☐	☐	☐	☐	☐	☐	☐
Persons with detected antibodies / tested persons (SERO-PREVALENCE)	☐	☐	☐	☐	☐	☐	☐

42. **If you selected "other" in the previous question, please specify here below**

____

43. **Is your country performing ROUTINELY VACCINATION FOR YELLOW FEVER?** *

Tick all that apply.

- [ ] YES
- [ ] NOT AT ALL
- [ ] NOT ROUTINELY / OTHER
- [ ] I DON'T KNOW

44. **If you selected "NOT ROUTINELY / OTHER" in the previous question, could you please specify here below?**

____

45. **If "YES" or "NOT ROUTINELY / OTHER", is your country collecting data on number of vaccinated per year or vaccination coverage, and at which level?**

Tick all that apply.

- [ ] NO
- [ ] YES - National aggregated
- [ ] YES - Regional aggregated
- [ ] YES - Local or municipal
- [ ] I don't know
- [ ] Other: ________________________________

46. **If "YES" or "NOT ROUTINELY / OTHER", where are the collected data on VACCINATION stored?**

Tick all that apply.

- [ ] Digitalized national database interoperable or integrated with other sectors' databases
- [ ] Digitalized national database
- [ ] Non digitalized national database
- [ ] Local or regional database
- [ ] I don't know
- [ ] Other: ________________________________

47. **Is your country collecting ANY OTHER RELEVANT INDICATOR not mentioned above? If yes, could you specify?** *

____

____

____

____

____

Rift Valley Fever virus

48. **Is Rift Valley Fever virus a pathogen of relevance for your country? (A relevant pathogen could be an endemic or epidemic pathogen in the country, or an emerging pathogen not yet identified in the country)** *

Mark only one oval.

- YES
- NO *Skip to question 53.*
- Other: ____________________

Rift Valley Fever virus

49. **Is your country collecting data on DISEASE FREQUENCY or OCCURRENCE and at which level?** *

Tick all that apply.

	No	YES (national aggregated)	YES (regional aggregated)	YES (local or GPS)	Other	I don't know
New notified cases / outbreaks (according to National case definition) per year	☐	☐	☐	☐	☐	☐
Number confirmed laboratory cases (according to National case definition) per year	☐	☐	☐	☐	☐	☐
Persons with detected antibodies / tested persons (SERO-PREVALENCE)	☐	☐	☐	☐	☐	☐

50. **If you selected "other" in the previous question, please specify here below**

____

51. **If yes, where are the collected data stored?**
Tick all that apply.

	Digitalized national database interoperable or integrated with other sectors' databases	Digitalized national database	Non digitalized national database	Local or regional database	Other	I don't know	Non applicable
New notified cases / outbreaks (according to National case definition) per year	☐	☐	☐	☐	☐	☐	☐
Number confirmed laboratory cases (according to National case definition) per year	☐	☐	☐	☐	☐	☐	☐
Persons with detected antibodies / tested persons (SERO-PREVALENCE)	☐	☐	☐	☐	☐	☐	☐

52. **If you selected "other" in the previous question, please specify here below**

53. **Is your country collecting ANY OTHER RELEVANT INDICATOR not mentioned above? If yes, could you specify?** *

West Nile virus

54. **Is West Nile Fever virus a pathogen of relevance for your country? (A relevant pathogen could be an endemic or epidemic pathogen in the country, or an emerging pathogen not yet identified in the country)** *
Mark only one oval.

- ◯ YES
- ◯ NO *Skip to question 59.*
- ◯ Other: ____________

West Nile virus

55. **Is your country collecting data on DISEASE FREQUENCY or OCCURRENCE and at which level?** *

Tick all that apply.

	No	YES (national aggregated)	YES (regional aggregated)	YES (local or GPS)	Other	I don't know
New notified cases / outbreaks (according to National case definition) per year	☐	☐	☐	☐	☐	☐
Number confirmed laboratory cases (according to National case definition) per year	☐	☐	☐	☐	☐	☐
Persons with detected antibodies / tested persons (SERO-PREVALENCE)	☐	☐	☐	☐	☐	☐

56. **If you selected "other" in the previous question, please specify here below**

57. **If yes, where are the collected data stored?**

Tick all that apply.

	Digitalized national database interoperable or integrated with other sectors' databases	Digitalized national database	Non digitalized national database	Local or regional database	Other	I don't know	Non applicable
New notified cases / outbreaks (according to National case definition) per year	☐	☐	☐	☐	☐	☐	☐
Number confirmed laboratory cases (according to National case definition) per year	☐	☐	☐	☐	☐	☐	☐
Persons with detected antibodies / tested persons (SERO-PREVALENCE)	☐	☐	☐	☐	☐	☐	☐

58. **If you selected "other" in the previous question, please specify here below**

59. **Is your country collecting ANY OTHER RELEVANT INDICATOR not mentioned above? If yes, could you specify?** *

Zika virus

60. **Is Zika virus a pathogen of relevance for your country? (A relevant pathogen could be an endemic or epidemic pathogen in the country, or an emerging pathogen not yet identified in the country)** *

Mark only one oval.

- YES
- NO *Skip to question 65.*
- Other:

Zika virus

61. **Is your country collecting data on DISEASE FREQUENCY or OCCURRENCE and at which level?** *

Tick all that apply.

	No	YES (national aggregated)	YES (regional aggregated)	YES (local or GPS)	Other	I don't know
New notified cases / outbreaks (according to National case definition) per year	☐	☐	☐	☐	☐	☐
Number confirmed laboratory cases (according to National case definition) per year	☐	☐	☐	☐	☐	☐
Persons with detected antibodies / tested persons (SERO-PREVALENCE)	☐	☐	☐	☐	☐	☐

62. **If you selected "other" in the previous question, please specify here below**

63. **If yes, where are the collected data stored?**
Tick all that apply.

	Digitalized national database interoperable or integrated with other sectors' databases	Digitalized national database	Non digitalized national database	Local or regional database	Other	I don't know	Non applicable
New notified cases / outbreaks (according to National case definition) per year	☐	☐	☐	☐	☐	☐	☐
Number confirmed laboratory cases (according to National case definition) per year	☐	☐	☐	☐	☐	☐	☐
Persons with detected antibodies / tested persons (SERO-PREVALENCE)	☐	☐	☐	☐	☐	☐	☐

64. **If you selected "other" in the previous question, please specify here below**

65. **Is your country collecting ANY OTHER RELEVANT INDICATOR not mentioned above? If yes, could you specify?** *

Climate & Environment

This section is collecting information on indicators regarding Climate & Environment regardless of the pathogens

Climate & Environment

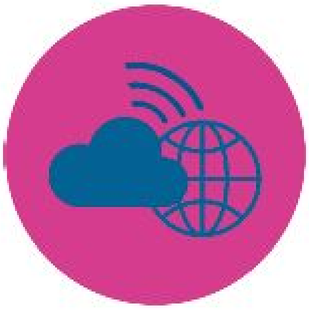

66. **Is your country collecting data on TEMPERATURE and at which level?** *
Tick all that apply.

- [ ] NO
- [ ] YES - National aggregated
- [ ] YES - Regional aggregated
- [ ] YES - Local or GPS
- [ ] I don't know
- [ ] Other: ____________________

67. **If yes, which specific data on TEMPERATURE is your country collecting?**
Tick all that apply.

- [ ] Maximum temperature
- [ ] Annual average of mean temperature
- [ ] I don't know
- [ ] Other: ____________________

68. **If yes, where are the collected data on TEMPERATURE stored?**
Tick all that apply.

- [ ] Digitalized national database interoperable or integrated with other sectors' databases
- [ ] Digitalized national database
- [ ] Non digitalized national database
- [ ] Local or regional database
- [ ] I don't know
- [ ] Other: ____________________

69. **Is your country collecting data on PRECIPITATIONS and at which level?** *
Tick all that apply.

- [ ] NO
- [ ] YES - National aggregated
- [ ] YES - Regional aggregated
- [ ] YES - Local or GPS
- [ ] I don't know
- [ ] Other: ____________________

70. **If yes, which specific data on PRECIPITATIONS is your country collecting?**
Tick all that apply.

- [ ] Monthly precipitation
- [ ] Weekly precipitation
- [ ] I don't know
- [ ] Other: ____________________

71. **If yes, where are the collected data on PRECIPITATIONS stored?**
Tick all that apply.

- [ ] Digitalized national database interoperable or integrated with other sectors' databases
- [ ] Digitalized national database
- [ ] Non digitalized national database
- [ ] Local or regional database
- [ ] I don't know
- [ ] Other: ____________________

72. **Where are the METEOROLOGICAL STATIONS of your country? Please specify place and region. If you don't know, please answer "I don't know".** *

____
____
____
____
____

73. **Which variables are routinely recorded in your meteorological stations?**
Tick all that apply.

- [ ] Temperature
- [ ] Precipitations
- [ ] I don't know
- [ ] Other: ____________________

74. **Is your country collecting data on VEGETATION and at which level?** *
Tick all that apply.

- [ ] NO
- [ ] YES - National aggregated
- [ ] YES - Regional aggregated
- [ ] YES - Local or GPS
- [ ] I don't know
- [ ] Other: ____________________

75. **If yes, which specific data on VEGETATION is your country collecting?**

____

76. **If yes, where are the collected data on VEGETATION stored?**
Tick all that apply.

- [ ] Digitalized national database interoperable or integrated with other sectors' databases
- [ ] Digitalized national database
- [ ] Non digitalized national database
- [ ] Local or regional database
- [ ] I don't know
- [ ] Other: ____________________

77. **Is your country collecting data on LAND USE and at which level?** *
Tick all that apply.

- [ ] NO
- [ ] YES - National aggregated
- [ ] YES - Regional aggregated
- [ ] YES - Local or GPS
- [ ] I don't know
- [ ] Other: ____________________

78. **If yes, which specific data on LAND USE is your country collecting?**
Tick all that apply.

- [ ] Forest, mixed vegetation, cropland or urban land uses
- [ ] I don't know
- [ ] Other: ____________________

79. **If yes, where are the collected data on LAND USE stored?**
Tick all that apply.

- [ ] Digitalized national database interoperable or integrated with other sectors' databases
- [ ] Digitalized national database
- [ ] Non digitalized national database
- [ ] Local or regional database
- [ ] I don't know
- [ ] Other: ____________________

80. **Is your country collecting data on LAND COVER and at which level?** *
Tick all that apply.

- [ ] NO
- [ ] YES - National aggregated
- [ ] YES - Regional aggregated
- [ ] YES - Local or GPS
- [ ] I don't know
- [ ] Other: ____________________

81. **If yes, which specific data on LAND COVER is your country collecting?**
Tick all that apply.

- [ ] Artificial, cultivated, herbaceous cover, tree cover, mosaic and water
- [ ] I don't know
- [ ] Other: ____________________

82. **If yes, where are the collected data on LAND COVER stored?**
Tick all that apply.

- [ ] Digitalized national database interoperable or integrated with other sectors’ databases
- [ ] Digitalized national database
- [ ] Non digitalized national database
- [ ] Local or regional database
- [ ] I don't know
- [ ] Other: ____________________

83. **Is your country collecting data on SOIL TYPE and at which level?** *
Tick all that apply.

- [ ] NO
- [ ] YES - National aggregated
- [ ] YES - Regional aggregated
- [ ] YES - Local or GPS
- [ ] I don't know
- [ ] Other: ____________________

84. **If yes, which specific data on SOIL TYPE is your country collecting?**

____

85. **If yes, where are the collected data on SOIL TYPE stored?**
Tick all that apply.

- [ ] Digitalized national database interoperable or integrated with other sectors’ databases
- [ ] Digitalized national database
- [ ] Non digitalized national database
- [ ] Local or regional database
- [ ] I don't know
- [ ] Other: ____________________

86. **The above mentioned indicators are collected by which INSTITUTION / INSTITUTIONS? If you don't know, please answer "I don't know".** *

____

____

____

____

____

87. **Has your office access to any GLOBAL PUBLIC DATASET related to Climate & Environment?** *

Mark only one oval.

- Yes
- No
- I don't know
- Other: ________________

88. **If yes, which one/ones? Are you using it/them for which purpose?**

89. **Is your country collecting ANY OTHER RELEVANT INDICATOR not mentioned above? If yes, could you specify?** *

Conclusions

90. **Please feel free to put any comment, suggestion or remark here below**

Thank you very much for your time and consideration!

MediLabSecure 2 is a project funded by the EC DEVCO (IFS/2018/402-247)

CBRN Centres of Excellence

An initiative of the European Union

Please be kindly informed that the information you have provided with this questionnaire will be shared and disseminated only in aggregated form with the information provided by all the other MLS Countries involved in this survey.

- [ ] Send me a copy of my responses.

Powered by
Google Forms
