# Supplementary material for: Integrated Early Warning Surveillance: Achilles′ Heel of One Health?
Source: Microorganisms. 2020 Jan 8;8(1):84. doi: 10.3390/microorganisms8010084 (PMC7022449; doi:10.3390/microorganisms8010084)
Supplement: Supplementary file 1 [file microorganisms-08-00084-s001.zip › Supplementary_Mat3_QuestionnaireAnimal.pdf]

Indicators for Early Warning and Risk Assessment - ANIMAL

Contact information

*Required

1. **Email address** *

MediLabSecure (MLS) One Health Project - Surveillance of Arbovirus infections

Purpose of the survey

Goal of this survey is to collect information on important indicators for risk assessment and early warning of arboviruses of relevance in the countries of the MLS Network, focusing on IF and HOW such information are collected at country level.

Please, complete each part of the questionnaire in the most accurate way possible.
For any further clarification, feel free to contact us at laura.amato@iss.it

Many thanks in advance for your time and consideration.

The Public Health Work Package of MLS

Structure of the questionnaire

The present questionnaire focuses on 7 relevant pathogens (namely Chikungunya virus, Crimean-Congo Haemorragic fever virus, Dengue fever virus, Yellow fever virus, Rift Valley fever virus, West Nile virus, Zika virus).

Each sector involved in the surveillance activities of the above pathogens (vector, human, animal) collects data in accordance with respective surveillance priorities. You are kindly asked to fill in this questionnaire that is specifically related to your field of expertise and sector of activity.

Each section is dedicated to one pathogen. At the beginning of each section, a question asking on the relevance of the pathogen for your country lets you provide information on the pathogen or skip to the next one.

Good luck and thank you for your support!

Animal

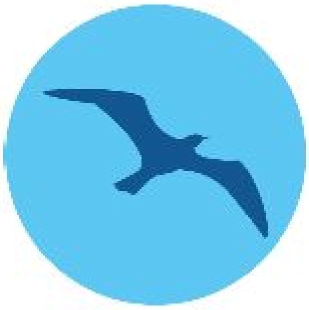

2. **Country** *

___

3. **Family name** *

___

4. **Given name** *

___

5. **Name of your Institution** *

___

6. **Name of your Laboratory** *

___

7. **Of which MediLabSecure networks is your laboratory part of?** *
Tick all that apply.

- [ ] Human Virology
- [ ] Animal Virology
- [ ] Medical Entomology
- [ ] Human Public Health
- [ ] Animal Public Health
- [ ] Other: ___

8. **Family name, given name and affiliation of other contributors to the compilation of the survey, if any**

___
___
___
___
___

Animal Health - General Section

9. **Is your country collecting data on ANIMAL POPULATION DENSITY and at which level?** *

Tick all that apply.

	No	YES (national aggregated)	YES (regional aggregated)	YES (local or municipal)	Other	I don't know
Cattle						
Goats						
Sheep						
Equids						
Camels						
Wild ruminants						
Wild ungulates other than ruminants						
Wild birds						

10. **If you selected "other" in the previous question, please specify here below**

11. **If yes, where are the collected data on ANIMAL POPULATION DENSITY stored?**

Tick all that apply.

	Digitalized national database interoperable or integrated with other sectors' databases	Digitalized national database	Non digitalized national database	Local or regional database	Other	I don't know
Cattle						
Goats						
Sheep						
Equids						
Camels						
Wild ruminants						
Wild ungulates other than ruminants						
Wild birds						

12. **If you selected "other" in the previous question, please specify here below**

13. **Is your country collecting data on ANIMAL MOVEMENTS AND TRADE and at which level?** *

Tick all that apply.

	No	YES (national aggregated)	YES (regional aggregated)	YES (local or GPS)	Other	I don't know
Pastoralism and transhumance						
Import and export						
Wildlife migrations						

14. **If you selected "other" in the previous question, please specify here below**

15. **If yes, where are the collected data on ANIMAL MOVEMENTS AND TRADE stored?**
Tick all that apply.

	Digitalized national database interoperable or integrated with other sectors' databases	Digitalized national database	Non digitalized national database	Local or regional database	Other	I don't know
Pastoralism and transhumance	☐	☐	☐	☐	☐	☐
Import and export	☐	☐	☐	☐	☐	☐
Wildlife migrations	☐	☐	☐	☐	☐	☐

16. **If you selected "other" in the previous question, please specify here below**

17. **Has your office access to any GLOBAL PUBLIC DATASET related to animals?** *
Mark only one oval.

- Yes
- No
- I don't know
- Other:

18. **If yes, which one/ones? Are you using it/them for which purpose?**

Crimean-Congo Haemorragic fever virus

19. **Is Crimean-Congo Haemorragic fever virus a pathogen of relevance for your country? (A relevant pathogen could be an endemic or epidemic pathogen in the country, or an emerging pathogen not yet identified in the country)** *
Mark only one oval.

- YES
- NO *Skip to question 28.*
- Other:

Crimean-Congo Haemorragic fever virus

20. **Is your country collecting data on ANIMAL DISEASE OCCURRENCE and at which level? (DISEASE OCCURRENCE: Notified cases / outbreaks (according to National case definition) per year) ***
Tick all that apply.

	No	YES (national aggregated)	YES (regional aggregated)	YES (local or GPS)	Other	I don't know
Cattle						
Goats						
Sheep						
Camels						
Wild ruminants						
Wild ungulates other than ruminants						
Wild birds						

21. **If you selected "other" in the previous question, please specify here below**

22. **If yes, where are the collected data on ANIMAL DISEASE OCCURRENCE stored?**
Tick all that apply.

	Digitalized national database interoperable or integrated with other sectors' databases	Digitalized national database	Non digitalized national database	Local or regional database	Other	I don't know
Cattle						
Goats						
Sheep						
Camels						
Wild ruminants						
Wild ungulates other than ruminants						
Wild birds						

23. **If you selected "other" in the previous question, please specify here below**

24. **Is your country collecting data on ANIMAL DISEASE SERO-PREVALENCE and at which level? (DISEASE SERO-PREVALENCE: Animals with detected antibodies / tested animals)** *

Tick all that apply.

	No	YES (national aggregated)	YES (regional aggregated)	YES (local or GPS)	Other	I don't know
Cattle						
Goats						
Sheep						
Camels						
Wild ruminants						
Wild ungulates other than ruminants						
Wild birds						

25. **If you selected "other" in the previous question, please specify here below**

26. **If yes, where are the collected data on ANIMAL DISEASE SERO-PREVALENCE stored?**

Tick all that apply.

	Digitalized national database interoperable or integrated with other sectors' databases	Digitalized national database	Non digitalized national database	Local or regional database	Other	I don't know
Cattle						
Goats						
Sheep						
Camels						
Wild ruminants						
Wild ungulates other than ruminants						
Wild birds						

27. **If you selected "other" in the previous question, please specify here below**

28. **Is your country collecting ANY OTHER RELEVANT INDICATOR not mentioned above? If yes, could you specify?** *

Rift Valley Fever virus

29. **Is Rift Valley Fever virus a pathogen of relevance for your country? (A relevant pathogen could be an endemic or epidemic pathogen in the country, or an emerging pathogen not yet identified in the country)** *

Mark only one oval.

- ( ) YES
- ( ) NO *Skip to question 44.*
- ( ) Other: ______________________

Rift Valley Fever virus

30. **Is your country collecting data on ANIMAL DISEASE OCCURRENCE and at which level? (DISEASE OCCURRENCE: Notified cases / outbreaks (according to National case definition) per year)** *

Tick all that apply.

	No	YES (national aggregated)	YES (regional aggregated)	YES (local or GPS)	Other	I don't know
Cattle	[ ]	[ ]	[ ]	[ ]	[ ]	[ ]
Goats	[ ]	[ ]	[ ]	[ ]	[ ]	[ ]
Sheep	[ ]	[ ]	[ ]	[ ]	[ ]	[ ]
Camels	[ ]	[ ]	[ ]	[ ]	[ ]	[ ]
Wild ruminants	[ ]	[ ]	[ ]	[ ]	[ ]	[ ]
Wild ungulates other than ruminants	[ ]	[ ]	[ ]	[ ]	[ ]	[ ]

31. **If you selected "other" in the previous question, please specify here below**

____

32. **If yes, where are the collected data on ANIMAL DISEASE OCCURRENCE stored?**

Tick all that apply.

	Digitalized national database interoperable or integrated with other sectors’ databases	Digitalized national database	Non digitalized national database	Local or regional database	Other	I don't know
Cattle	[ ]	[ ]	[ ]	[ ]	[ ]	[ ]
Goats	[ ]	[ ]	[ ]	[ ]	[ ]	[ ]
Sheep	[ ]	[ ]	[ ]	[ ]	[ ]	[ ]
Camels	[ ]	[ ]	[ ]	[ ]	[ ]	[ ]
Wild ruminants	[ ]	[ ]	[ ]	[ ]	[ ]	[ ]
Wild ungulates other than ruminants	[ ]	[ ]	[ ]	[ ]	[ ]	[ ]

33. **If you selected "other" in the previous question, please specify here below**

____

34. **Is your country collecting data on ANIMAL DISEASE SERO-PREVALENCE and at which level? (DISEASE SERO-PREVALENCE: Animals with detected antibodies / tested animals)** *

Tick all that apply.

	No	YES (national aggregated)	YES (regional aggregated)	YES (local or GPS)	Other	I don't know
Cattle	☐	☐	☐	☐	☐	☐
Goats	☐	☐	☐	☐	☐	☐
Sheep	☐	☐	☐	☐	☐	☐
Camels	☐	☐	☐	☐	☐	☐
Wild ruminants	☐	☐	☐	☐	☐	☐
Wild ungulates other than ruminants	☐	☐	☐	☐	☐	☐

35. **If you selected "other" in the previous question, please specify here below**

____

36. **If yes, where are the collected data on ANIMAL DISEASE SERO-PREVALENCE stored?**

Tick all that apply.

	Digitalized national database interoperable or integrated with other sectors’ databases	Digitalized national database	Non digitalized national database	Local or regional database	Other	I don't know
Cattle	☐	☐	☐	☐	☐	☐
Goats	☐	☐	☐	☐	☐	☐
Sheep	☐	☐	☐	☐	☐	☐
Camels	☐	☐	☐	☐	☐	☐
Wild ruminants	☐	☐	☐	☐	☐	☐
Wild ungulates other than ruminants	☐	☐	☐	☐	☐	☐

37. **If you selected "other" in the previous question, please specify here below**

____

38. **Is your country ROUTINELY VACCINATING animals for the disease?** *

Mark only one oval per row.

	Yes	Not at all	Not routinely / others	I don't know
Cattle	◯	◯	◯	◯
Goats	◯	◯	◯	◯
Sheep	◯	◯	◯	◯
Camels	◯	◯	◯	◯
Wild ruminants	◯	◯	◯	◯
Wild ungulates other than ruminants	◯	◯	◯	◯

39. **If you selected "others" in the previous question, please specify here below**

____

40. **If yes, is your country collecting data on NUMBER OF VACCINATED ANIMALS PER YEAR OR VACCINATION COVERAGE and at which level?**
Tick all that apply.

	No	YES (national aggregated)	YES (regional aggregated)	YES (local or GPS)	Other	I don't know
Cattle	☐	☐	☐	☐	☐	☐
Goats	☐	☐	☐	☐	☐	☐
Sheep	☐	☐	☐	☐	☐	☐
Camels	☐	☐	☐	☐	☐	☐
Wild ruminants	☐	☐	☐	☐	☐	☐
Wild ungulates other than ruminants	☐	☐	☐	☐	☐	☐

41. **If you selected "other" in the previous question, please specify here below**

42. **If yes, where are the collected data on ANIMAL VACCINATION stored?**
Tick all that apply.

	Digitalized national database interoperable or integrated with other sectors' databases	Digitalized national database	Non digitalized national database	Local or regional database	Other	I don't know
Cattle	☐	☐	☐	☐	☐	☐
Goats	☐	☐	☐	☐	☐	☐
Sheep	☐	☐	☐	☐	☐	☐
Camels	☐	☐	☐	☐	☐	☐
Wild ruminants	☐	☐	☐	☐	☐	☐
Wild ungulates other than ruminants	☐	☐	☐	☐	☐	☐

43. **If you selected "other" in the previous question, please specify here below**

44. **Is your country collecting ANY OTHER RELEVANT INDICATOR not mentioned above? If yes, could you specify?** *

West Nile fever virus

45. **Is West Nile Fever virus a pathogen of relevance for your country? (A relevant pathogen could be an endemic or epidemic pathogen in the country, or an emerging pathogen not yet identified in the country)** *
Mark only one oval.

- YES
- NO *Skip to question 60.*
- Other: ____________

West Nile fever virus

46. **Is your country collecting data on ANIMAL DISEASE OCCURRENCE and at which level? (DISEASE OCCURRENCE: Notified cases / outbreaks (according to National case definition) per year)** *
Tick all that apply.

	No	YES (national aggregated)	YES (regional aggregated)	YES (local or GPS)	Other	I don't know
Equids						
Wild birds						

47. **If you selected "other" in the previous question, please specify here below**

____

48. **If yes, where are the collected data on ANIMAL DISEASE OCCURRENCE stored?**
Tick all that apply.

	Digitalized national database interoperable or integrated with other sectors' databases	Digitalized national database	Non digitalized national database	Local or regional database	Other	I don't know
Equids						
Wild birds						

49. **If you selected "other" in the previous question, please specify here below**

____

50. **Is your country collecting data on ANIMAL DISEASE SERO-PREVALENCE and at which level? (DISEASE SERO-PREVALENCE: Animals with detected antibodies / tested animals)** *
Tick all that apply.

	No	YES (national aggregated)	YES (regional aggregated)	YES (local or GPS)	Other	I don't know
Equids						
Wild birds						

51. **If you selected "other" in the previous question, please specify here below**

____

52. **If yes, where are the collected data on ANIMAL DISEASE SERO-PREVALENCE stored?**
Tick all that apply.

	Digitalized national database interoperable or integrated with other sectors' databases	Digitalized national database	Non digitalized national database	Local or regional database	Other	I don't know
Equids	☐	☐	☐	☐	☐	☐
Wild birds	☐	☐	☐	☐	☐	☐

53. **If you selected "other" in the previous question, please specify here below**

54. **Is your country ROUTINELY VACCINATING animals for the disease?** *
Mark only one oval per row.

	Yes	Not at all	Not routinely / others	I don't know
Equids	◯	◯	◯	◯
Others	◯	◯	◯	◯

55. **If you selected "others" in the previous question, please specify here below**

56. **If yes, is your country collecting data on NUMBER OF VACCINATED ANIMALS PER YEAR OR VACCINATION COVERAGE and at which level?**
Tick all that apply.

	No	YES (national aggregated)	YES (regional aggregated)	YES (local or GPS)	Other	I don't know
Equids	☐	☐	☐	☐	☐	☐
Others	☐	☐	☐	☐	☐	☐

57. **If you selected "other" in the previous question, please specify here below**

58. **If yes, where are the collected data on ANIMAL VACCINATION stored?**
Tick all that apply.

	Digitalized national database interoperable or integrated with other sectors' databases	Digitalized national database	Non digitalized national database	Local or regional database	Other	I don't know
Equids	☐	☐	☐	☐	☐	☐
Others	☐	☐	☐	☐	☐	☐

59. **If you selected "other" in the previous question, please specify here below**

60. **Is your country collecting ANY OTHER RELEVANT INDICATOR not mentioned above? If yes, could you specify?** *

Climate & Environment

This section is collecting information on indicators regarding Climate & Environment regardless of the pathogens

Climate & Environment

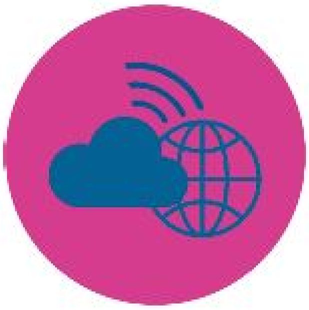

61. **Is your country collecting data on TEMPERATURE and at which level?** *

Tick all that apply.

- [ ] NO
- [ ] YES - National aggregated
- [ ] YES - Regional aggregated
- [ ] YES - Local or GPS
- [ ] I don't know
- [ ] Other:

62. **If yes, which specific data on TEMPERATURE is your country collecting?**

Tick all that apply.

- [ ] Maximum temperature
- [ ] Annual average of mean temperature
- [ ] I don't know
- [ ] Other:

63. **If yes, where are the collected data on TEMPERATURE stored?**
Tick all that apply.

- [ ] Digitalized national database interoperable or integrated with other sectors' databases
- [ ] Digitalized national database
- [ ] Non digitalized national database
- [ ] Local or regional database
- [ ] I don't know
- [ ] Other: ____________

64. **Is your country collecting data on PRECIPITATIONS and at which level?** *
Tick all that apply.

- [ ] NO
- [ ] YES - National aggregated
- [ ] YES - Regional aggregated
- [ ] YES - Local or GPS
- [ ] I don't know
- [ ] Other: ____________

65. **If yes, which specific data on PRECIPITATIONS is your country collecting?**
Tick all that apply.

- [ ] Monthly precipitation
- [ ] Weekly precipitation
- [ ] I don't know
- [ ] Other: ____________

66. **If yes, where are the collected data on PRECIPITATIONS stored?**
Tick all that apply.

- [ ] Digitalized national database interoperable or integrated with other sectors' databases
- [ ] Digitalized national database
- [ ] Non digitalized national database
- [ ] Local or regional database
- [ ] I don't know
- [ ] Other: ____________

67. **Where are the METEOROLOGICAL STATIONS of your country? Please specify place and region. If you don't know, please answer "I don't know".** *

____
____
____
____
____

68. **Which variables are routinely recorded in your meteorological stations?**
Tick all that apply.

- [ ] Temperature
- [ ] Precipitations
- [ ] I don't know
- [ ] Other: ____________________

69. **Is your country collecting data on VEGETATION and at which level?** *
Tick all that apply.

- [ ] NO
- [ ] YES - National aggregated
- [ ] YES - Regional aggregated
- [ ] YES - Local or GPS
- [ ] I don't know
- [ ] Other: ____________________

70. **If yes, which specific data on VEGETATION is your country collecting?**

____

71. **If yes, where are the collected data on VEGETATION stored?**
Tick all that apply.

- [ ] Digitalized national database interoperable or integrated with other sectors' databases
- [ ] Digitalized national database
- [ ] Non digitalized national database
- [ ] Local or regional database
- [ ] I don't know
- [ ] Other: ____________________

72. **Is your country collecting data on LAND USE and at which level?** *
Tick all that apply.

- [ ] NO
- [ ] YES - National aggregated
- [ ] YES - Regional aggregated
- [ ] YES - Local or GPS
- [ ] I don't know
- [ ] Other: ____________________

73. **If yes, which specific data on LAND USE is your country collecting?**
Tick all that apply.

- [ ] Forest, mixed vegetation, cropland or urban land uses
- [ ] I don't know
- [ ] Other: ____________________

74. **If yes, where are the collected data on LAND USE stored?**
Tick all that apply.

- [ ] Digitalized national database interoperable or integrated with other sectors’ databases
- [ ] Digitalized national database
- [ ] Non digitalized national database
- [ ] Local or regional database
- [ ] I don't know
- [ ] Other: ____________________

75. **Is your country collecting data on LAND COVER and at which level?** *
Tick all that apply.

- [ ] NO
- [ ] YES - National aggregated
- [ ] YES - Regional aggregated
- [ ] YES - Local or GPS
- [ ] I don't know
- [ ] Other: ____________________

76. **If yes, which specific data on LAND COVER is your country collecting?**
Tick all that apply.

- [ ] Artificial, cultivated, herbaceous cover, tree cover, mosaic and water
- [ ] I don't know
- [ ] Other: ____________________

77. **If yes, where are the collected data on LAND COVER stored?**
Tick all that apply.

- [ ] Digitalized national database interoperable or integrated with other sectors’ databases
- [ ] Digitalized national database
- [ ] Non digitalized national database
- [ ] Local or regional database
- [ ] I don't know
- [ ] Other: ____________________

78. **Is your country collecting data on SOIL TYPE and at which level?** *
Tick all that apply.

- [ ] NO
- [ ] YES - National aggregated
- [ ] YES - Regional aggregated
- [ ] YES - Local or GPS
- [ ] I don't know
- [ ] Other: ____________________

79. **If yes, which specific data on SOIL TYPE is your country collecting?**

____

80. **If yes, where are the collected data on SOIL TYPE stored?**
Tick all that apply.

- [ ] Digitalized national database interoperable or integrated with other sectors' databases
- [ ] Digitalized national database
- [ ] Non digitalized national database
- [ ] Local or regional database
- [ ] I don't know
- [ ] Other: ______________________________

81. **The above mentioned indicators are collected by which INSTITUTION / INSTITUTIONS? If you don't know, please answer "I don't know".** *

____
____
____
____
____

82. **Has your office access to any GLOBAL PUBLIC DATASET related to Climate & Environment?** *
Mark only one oval.

- ( ) Yes
- ( ) No
- ( ) I don't know
- ( ) Other: ______________________________

83. **If yes, which one/ones? Are you using it/them for which purpose?**

____
____
____
____
____

84. **Is your country collecting ANY OTHER RELEVANT INDICATOR not mentioned above? If yes, could you specify?** *

____
____
____
____
____

Conclusions

85. **Please feel free to put any comment, suggestion or remark here below**

Thank you very much for your time and consideration!

MediLabSecure 2 is a project funded by the EC DEVCO (IFS/2018/402-247)

CBRN
Centres of Excellence
An initiative of the European Union

Please be kindly informed that the information you have provided with this questionnaire will be shared and disseminated only in aggregated form with the information provided by all the other MLS Countries involved in this survey.

☐ Send me a copy of my responses.

Powered by
Google Forms
